# Senescent Tumor Cells Build a Cytokine Shield in Colorectal Cancer

**DOI:** 10.1002/advs.202002497

**Published:** 2021-01-04

**Authors:** Yong Won Choi, Young Hwa Kim, Seung Yeop Oh, Kwang Wook Suh, Young‐Sam Kim, Ga‐Yeon Lee, Jung Eun Yoon, Soon Sang Park, Young‐Kyoung Lee, Yoo Jung Park, Hong Seok Kim, So Hyun Park, Jang‐Hee Kim, Tae Jun Park

**Affiliations:** ^1^ Department of Biochemistry and Molecular Biology Ajou University School of Medicine Suwon 16499 Korea; ^2^ Department of Hematology–Oncology Ajou University School of Medicine Suwon 16499 Korea; ^3^ Inflamm‐Aging Translational Research Center Ajou University Medical Center Suwon 16499 Korea; ^4^ Department of Biomedical Sciences Ajou University Graduate School of Medicine Suwon 16499 Korea; ^5^ Department of Surgery Ajou University School of Medicine Suwon 16499 Korea; ^6^ Department of Molecular Medicine Inha University School of Medicine Incheon 22212 Korea; ^7^ Department of Pathology Ajou University School of Medicine Suwon 16499 Korea

**Keywords:** cancer immunotherapy, CD8^+^ T cells, colorectal cancers, CXCL12, senescent tumor cells

## Abstract

Cellular senescence can either support or inhibit cancer progression. Here, it is shown that intratumoral infiltration of CD8^+^ T cells is negatively associated with the proportion of senescent tumor cells in colorectal cancer (CRC). Gene expression analysis reveals increased expression of C‐X‐C motif chemokine ligand 12 (CXCL12) and colony stimulating factor 1 (CSF1) in senescent tumor cells. Senescent tumor cells inhibit CD8^+^ T cell infiltration by secreting a high concentration of CXCL12, which induces a loss of CXCR4 in T cells that result in impaired directional migration. CSF1 from senescent tumor cells enhance monocyte differentiation into M2 macrophages, which inhibit CD8^+^ T cell activation. Neutralization of CXCL12/CSF1 increases the effect of anti‐PD1 antibody in allograft tumors. Furthermore, inhibition of CXCL12 from senescent tumor cells enhances T cell infiltration and results in reducing the number and size of tumors in azoxymethane (AOM)/dextran sulfate sodium (DSS)‐induced CRC. These findings suggest senescent tumor cells generate a cytokine barrier protecting nonsenescent tumor cells from immune attack and provide a new target for overcoming the immunotherapy resistance of CRC.

## Graphical abstract

Cellular senescence can either support or inhibit cancer progression. In colorectal cancer, senescent tumor cells inhibit CD8^+^ T cell infiltration by secreting a high concentration of CXCL12 and enhanced M2 macrophage differentiation by CSF1. Neutralization of CXCL12/CSF1 increases the effect of anti‐PD1 antibody. These findings suggest that senescent tumor cells generate a cytokine barrier protecting nonsenescent tumor cells from immune attack.

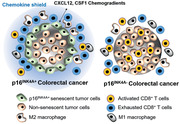

## 1 Introduction

Cellular senescence, a state of irreversible cell cycle arrest in response to diverse stimuli, including telomere attrition, genotoxic damage, and oncogene activation, is historically considered to be an essential anticarcinogenic barrier in normal cells.^[^ ^1^, ^2^, ^3^ ^]^ Nonetheless, senescent tumor cells have been found not only in premalignant tumors but also in developed malignant tumors.^[^ ^4^ ^]^ Although several studies have suggested that senescent stromal fibroblasts could promote the proliferative and metastatic properties of adjacent tumor cells^[^ ^5^, ^6^ ^]^ through the senescence‐associated secretory phenotype (SASP),^[^ ^7^, ^8^ ^]^ whether naturally occurring senescent tumor cells in malignant tumors could mediate similar protumorigenic effects has not yet been fully elucidated. In our previous study, senescent tumor cells were shown to play an important role in cancer progression: senescent tumor cells are actively involved in the collective invasion and metastasis via CXCL12/CXCR4 signaling.^[^ ^9^ ^]^ In subsequent studies, we found that immune cells could not infiltrate tumor tissues and were located around senescent tumor cells in colorectal cancer (CRC). This observation led to the hypothesis that senescent tumor cells play a role in inhibiting intratumoral immune cell infiltration.

Advanced CRC remains incurable despite therapeutic improvements like the incorporation of targeted therapy into chemotherapy.^[^ ^10^ ^]^ Although immune checkpoint inhibitors (ICIs) have dramatically changed the landscape of therapeutics for a variety of malignancies,^[^ ^11^ ^]^ microsatellite stable (MSS) CRC has not shown similar beneficial efficacy.^[^ ^12^, ^13^ ^]^ Nonetheless, in CRC, the type, location, and density of immune cells (including CD8^+^ T cells^[^ ^14^ ^]^) is represented by the “Immunoscore,”^[^ ^15^ ^]^ which has been validated as a better prognostic factor than the traditional TNM staging system and may be as a stronger predictor of patient survival than microsatellite instability (MSI).^[^ ^16^ ^]^ Therefore, the discovery of the intrinsic antitumor immune mechanism of CRC is critical to improving the efficacy of immunotherapy.^[^ ^17^ ^]^

For that reason, we postulate that our observations are closely related to the limited efficacy of immunotherapy in CRC, and we hypothesize that senescent tumor cells can inhibit immune cell infiltration. To explore this hypothesis, we analyze the distribution of immune cells, including CD8^+^ T cells and macrophages, and we examine the role of senescent tumor cells in immune cell function in MSS CRC.

## 2 Results

### 2.1 Senescent Tumor Cells in CRC Are Associated with Immune Reactions

**Figure 1:**
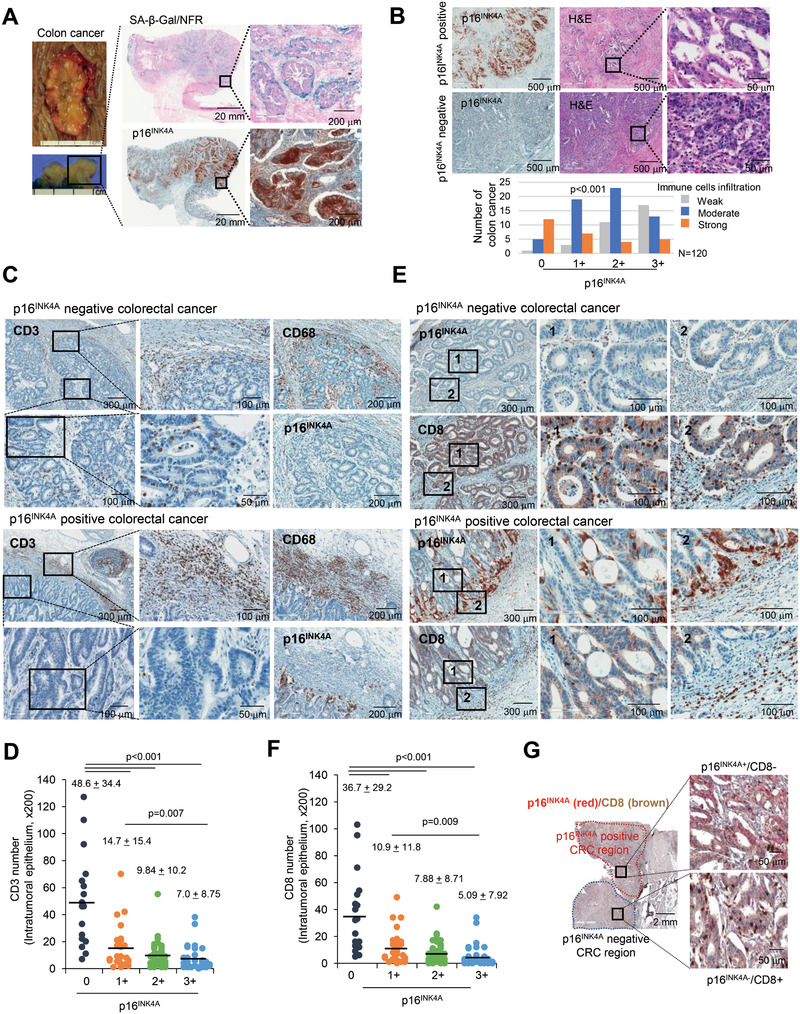
Senescent tumor cells are frequently identified in CRC. A) Fresh CRC tissues were divided into two identical tissue sections and processed for either fresh frozen section for SA‐*β*‐Gal staining or as FFPE section for p16^INK4A^ immunostaining. Nuclear fast red (NFR) for SA‐*β*‐Gal staining was applied as counterstain. The upper left panel shows the gross appearance of CRC, and the lower left panel shows the cross section of the CRC. The upper and lower right panels show the results of SA‐*β*‐Gal/NFR and p16^INK4A^ immunostaining, respectively. B) *χ* ^2^ analysis of immune cell infiltration according to grades of p16^INK4A^ immunostaining in 120 cases of MSS CRC tissues. C) CD3 positive cells infiltrated into p16^INK4A^ negative CRC. p16^INK4A^ negative and positive CRC tissues were serially dissected and immunostained for CD3, p16^INK4A^ and CD68. D) Infiltrated CD3^+^ T cell numbers were analyzed according to the grades of p16^INK4A^ (200× field). E) CD8 and p16^INK4A^ immunostaining in CRC. “1” and “2” indicate the high magnification views of the original figure. F) Infiltrated CD8^+^ T cell numbers were analyzed according to the grades of p16^INK4A^ (200× field). G) CRC tissues were stained with p16^INK4A^ and CD8. The upper p16^INK4A^ positive area and the lower p16^INK4A^ negative area of the cancer showed different patterns of CD8^+^ T cell infiltration. p16^INK4A^ negative and positive CRC indicates grade 0 and 1+, 2+, and 3+, respectively. The *p* value (D, F) was calculated by one‐way ANOVA and post hoc analysis. Results are presented as mean ± SD.

To investigate senescent tumor cells in CRC, we performed senescence‐associated beta‐galactosidase (SA‐*β*‐Gal) staining, a standard marker of cellular senescence, and found that the majority of SA‐*β*‐Gal‐positive cells were identified in the tumor epithelial areas of CRC. However, isolated round SA‐*β*‐Gal‐positive cells were also scattered in the stroma adjacent to the cancer cells (**Figure** **1**A; Figure S1A, Supporting Information). Because there are several limitations to the use of fresh tissues obtained during surgery, formalin‐fixed‐paraffin‐embedded (FFPE) tissues were used to identify senescent tumor cells. p16^INK4A^ expression has been validated as the most representative marker for in vivo detection of senescent cells^[^ ^18^ ^]^ and can be used to track^[^ ^19^ ^]^ and eliminate^[^ ^20^ ^]^ senescent cells in genetically engineered mouse models. Therefore, we performed p16^INK4A^ immunostaining using 27 cases of FFPE tissue sections, which were identical tissues to corresponding cases of SA‐*β*‐Gal staining and found that p16^INK4A^ immunopositivity was correlated with SA‐*β*‐Gal positivity (Figure 1A; *N* = 27, kappa = 0.598, *p* < 0.001). Since senescence is a state of irreversible growth arrest, most of the p16^INK4A^ positive tumor cells were negative for Ki67 immunostaining, indicating senescence (Figure S1B, Supporting Information). We further confirmed cellular senescence using another senescent marker, histone H3K9me^3^ (H3K9 trimethyl) (Figure S1C, Supporting Information).^[^ ^21^ ^]^ Moreover, p16^INK4A^ positive cells were positive for CDX2 immunostaining but negative for vimentin, indicating that these cells were not stromal cells but rather cancer epithelial cells (Figure S1D,E, Supporting Information). To examine whether a clinicopathological association exists between the senescent tumor cells and CRC, we classified 130 CRC cases into 4 grades according to the proportion of p16^INK4A^ (0: less than 1%; 1+: 1–20%; 2+: 20–40%; and 3+: more than 40% of cancer cells). Similar to our previous study,^[^ ^9^ ^]^ p16^INK4A^ immunostaining was found more frequently in the invasive region, and this finding is correlated with an advanced nodal stage (pN2, *p* = 0.011). Interestingly, intratumoral immune cell infiltration was significantly associated with the grades of p16^INK4A^ immunostaining (*p* < 0.001) (Table S1, Supporting Information). The immune cell infiltration in CRC is possibly associated with MSI‐related high mutational loads.^[^ ^22^ ^]^ In addition, mucinous carcinoma has been found to be associated with the presence of MSI and immune cell infiltration.^[^ ^23^ ^]^ To exclude the association of immune cell infiltration by MSI positive CRC or mucinous CRC and to elucidate the role of p16^INK4A^ positive senescent tumor cells in immune infiltration, we excluded ten cases of MSI positive and mucinous cancers. More than 60% of cancers showed grade 2 or 3 of p16^INK4A^ immunostaining (Figure S1F, Supporting Information). Although ten cases were excluded, the intratumoral immune cell infiltration was still significantly associated with the grades of p16^INK4A^ immunostaining in MSS CRC (*p* < 0.001) (Figure 1B; Table S1, Supporting Information). In terms of immune cell infiltrations, the p16^INK4A^ negative MSS CRC showed frequent intratumoral CD45^+^ immune cell infiltration compared to the p16^INK4A^ positive tumors (Figure S2A, Supporting Information). To prove the relationship between p16^INK4A^ expression and immune cell infiltration, we immunostained with CD3 and CD8 for T cell markers and CD68 for the monocyte and macrophage marker in 120 cases of MSS CRC with p16^INK4A^. The immunohistochemical analysis results revealed a marked accumulation of CD68 and CD3 positive cells around the invasive margin of the p16^INK4A^ positive cancer. By contrast, diffuse intratumoral infiltration of CD3^+^ T cells was observed in the p16^INK4A^ negative cancer. As for the CD68^+^ cells, various patterns of infiltration were observed (Figure 1C,D). We also evaluated the infiltration of CD8^+^ and CD4^+^ T cells; interestingly, the intratumoral infiltration of CD8^+^ T cells was markedly upregulated in p16^INK4A^ negative cancer but was rarely identified in p16^INK4A^ positive cancer. In addition, CD8^+^ T cells frequently accumulated in the invasive front of the p16^INK4A^ positive cancer (Figure 1E). Intratumoral CD8^+^ T cell infiltration varied significantly according to the proportion of p16^INK4A^ positive tumor cells with a reverse correlation (Figure 1F). Infiltrated T cells showed CD3/CD8 double positive (Figure S2B, Supporting Information). In addition, CD8^+^ T cell infiltration was markedly different, even in areas of the same tumors, dependent upon the presence or absence of p16^INK4A^ positive senescent tumor cells (Figure 1G). Unlike CD8^+^ T cells, CD4^+^ T cells were observed to be located only in the cancer margin with no invasion into the tumor epithelium in p16^INK4A^ negative CRC (Figure S2C, Supporting Information). FoxP3^+^ regulatory T cells also did not show infiltration in the intratumoral region in either group, and there were no differences in the distribution between p16^INK4A^ positive and negative CRC (Figure S2D, Supporting Information). These findings suggest that intratumoral infiltration of CD8^+^ T cells is inhibited in p16^INK4A^ positive CRC.

### 2.2 Senescent Tumor Cells Inhibit Intratumoral CD8^+^ T Cell Infiltration

**Figure 2:**
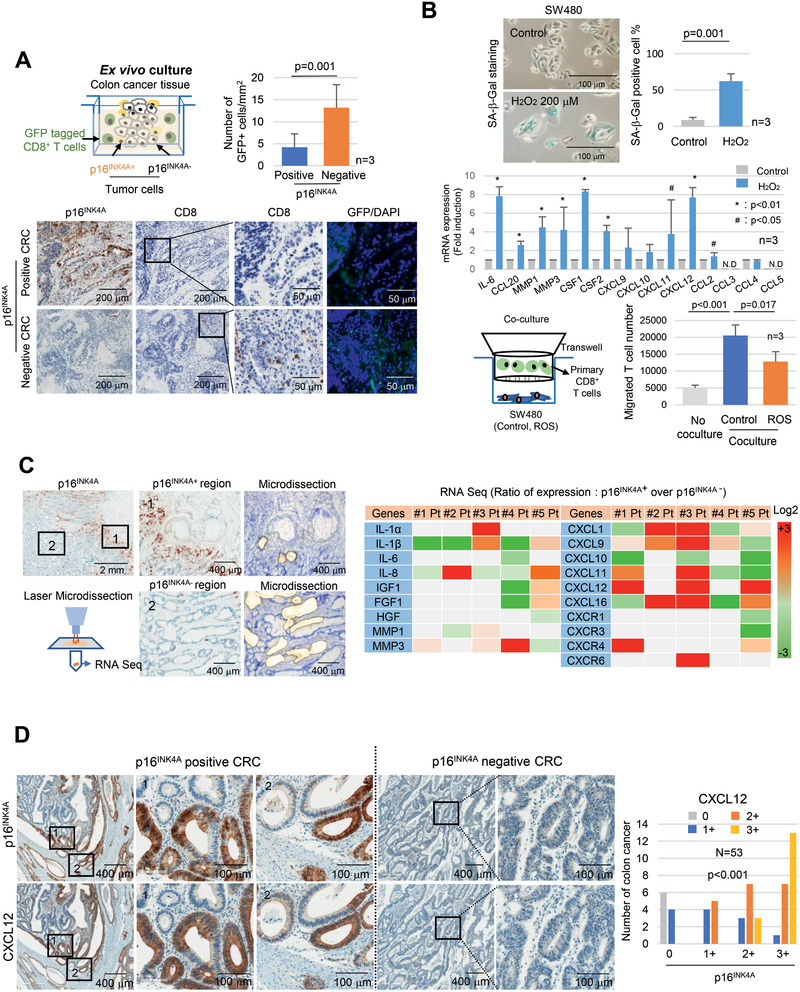
Senescent tumor cells exclude CD8^+^ T cells. A) Ex vivo culture. p16^INK4A^ positive or negative CRC tissues were cocultured with the GFP lentivirus infected isolated primary CD8^+^ T cells for 24 h and then stained with p16^INK4A^, CD8, and GFP. The number of GFP positive cells was counted and presented as a bar graph. B) Senescent tumor cells inhibited CD8^+^ T cell migration. SW480 cells were treated with H_2_O_2_ (200 × 10^−6^ m) for 3 days and analyzed for SA‐*β*‐Gal expression (upper panel) and SASP expression (middle panel). T cell migration assay. Isolated primary CD8^+^ T cells were cocultured with SW480 (control or H_2_O_2_ treated) for 3 h, and the number of migrated CD8^+^ T cells was counted. C) Microdissection analysis. CRC tissues were serially dissected and stained with p16^INK4A^ and toluidine blue. p16^INK4A^ positive and negative regions were microdissected and then analyzed for mRNA expression (*N* = 5, upper panel). The expression in RNA sequencing indicates the relative values of the p16^INK4A^ positive region compared with those of the p16^INK4A^ negative region. D) CXCL12 expression in p16^INK4A^ expressing senescent tumor cells. p16^INK4A^ positive and negative CRC tissues were serially dissected and stained with p16^INK4A^ and CXCL12 antibody, respectively. “1” and “2” indicate the high magnification views of the original figure. Results are presented as mean ± SD. The *p* value was calculated by the Mann–Whitney *U* test A) or Kruskall–Wallis test B) or *χ* ^2^ test D). *N* and *n* indicated the number of cases and independent experiments, respectively.

Next, we investigated the role of senescent tumor cells in CD8^+^ T cell infiltration. We hypothesized that senescent tumor cells secrete SASP that hinders the directional movement of the CD8^+^ T cell toward tumor nest. Ex vivo culture analysis revealed the infiltration of exogenous added primary CD8^+^ T cells in p16^INK4A^ negative cancer tissues, whereas exogenous CD8^+^ T cell infiltration was rarely present in the p16^INK4A^ positive cancer (**Figure** **2**A). To gain further insight into the negative effects of senescent tumor cells on the infiltration of CD8^+^ T cells, we applied in vitro senescent tumor cell models. The main inducer of tumor cell senescence in CRC has not yet been clearly established. Both chemotherapy and radiotherapy can cause senescence by DNA damage. However, in the present cohort, cases treated with either type were excluded. The oncogenes activation, such as RAS or BRAF, and the tumor suppressors inactivation, such as TP53 or APC, can induce oncogene‐induced senescence. However, there is not a significant association between oncogene activation or tumor suppressor gene inactivation and the presence of senescent tumor cells (Table S2, Supporting Information). Regardless of such genetic alterations, senescent tumor cells were more frequently identified at the invasive front of CRC, which has been found to be associated with hypoxia.^[^ ^24^ ^]^ Since oxygenation after hypoxia stimulates mitochondrial reactive oxygen species (ROS) production,^[^ ^25^ ^]^ and because ROS can lead to senescence, the in vitro ROS induced senescent tumor cells model was applied. ROS induced senescent tumor cells (SW480 colon cancer cells) produce several kinds of SASP. In a transwell migration assay, SW480 cells induced CD8^+^ T cell migration toward the cancer cells; however, ROS induced senescent SW480 cells inhibited the migration of CD8^+^ T cells (Figure 2B). CD8^+^ T lymphocyte infiltration is tightly regulated by chemotactic attractants.^[^ ^26^ ^]^ To more precisely investigate chemokine expression in senescent tumor cells, we compared the gene expression profiles of the p16^INK4A^ positive and negative tumor cells that were isolated by a laser microdissector (Figure 2C) through RNA sequencing. Among these molecules, we focused on T cell chemokines and specifically on CXC ligands (CXCLs). We found that CXCL12 was upregulated in p16^INK4A^ positive senescent tumor cells in three out of five patients (Figure 2C). The full RNA sequencing data are available at GEO (GSE125253). The expression of CXCL9, CXCL10, CXCL11, CXCL12, and CXCL16 were further analyzed by immunohistochemical staining; the results showed that CXCL9, CXCL10, CXCL11, and CXCL16 were not or expressed weakly in CRC cells and were only occasionally expressed in stromal cells (Figure S3A, Supporting Information), but CXCL12 was expressed in the areas where p16^INK4A^ positive tumor cells were found. We further evaluated CXCL12 expression in CRC through immunohistochemical staining in p16^INK4A^ positive CRC and found that CXCL12 expression was highly correlated with p16^INK4A^ expression (Figure 2D; Figure S3B, Supporting Information). CXCL12 expression was observed in 83.4 ± 13.9% of p16^INK4A^ expressing cells. On the other hand, CXCR4, the receptor of CXCL12, showed heterogenous expression patterns and its expression was not correlated with the existence of senescent tumor cells in CRC tissues (Figure S3C,D, Supporting Information). Furthermore, CXCR4 expression was also observed in stromal area (Figure S3D, Supporting Information).

### 2.3 High CXCL12 Concentrations Inhibit CD8^+^ T Cell Chemotaxis In Vitro and In Vivo

**Figure 3:**
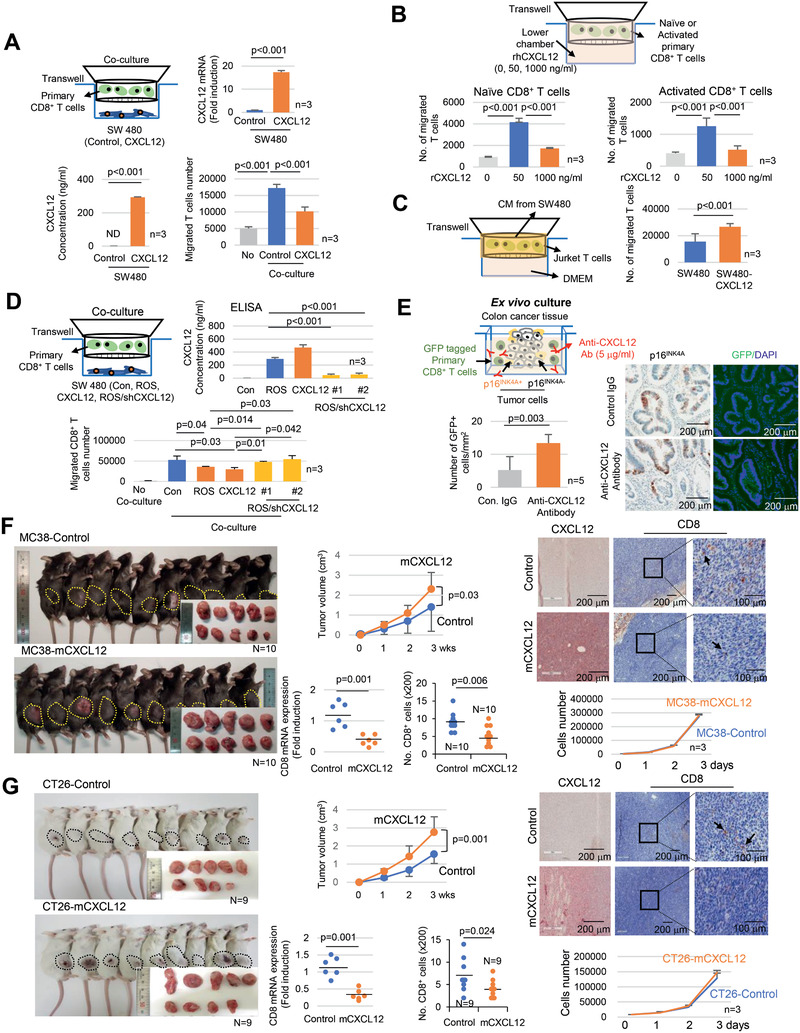
CXCL12 excludes CD8^+^ T cells. A) Isolated primary CD8^+^ T cells were cocultured with SW480 (control or CXCL12 overexpressed) for 3 h, and T cell migration was analyzed (lower right panel). B) The lower chambers of transwell were treated with 0, 50, and 1000 ng mL^−1^ rhCXCL12 and cultured with isolated primary naïve or CD3/CD28 activated CD8^+^ T cells for 3 h, T cell migration was subsequently analyzed. C) Jurkat T cells were cultured with the CM from SW480 cells (control or CXCL12 overexpressing cells) for 3 h and then T cell migration was analyzed. D) Isolated primary CD8^+^ T cells were cocultured with SW480 (control, ROS treated, CXCL12 overexpressing or ROS/shCXCL12) for 3 h and then T cell migration was analyzed. E) Ex vivo culture. p16^INK4A^ positive CRC tissues were cocultured with GFP lentivirus infected isolated primary CD8^+^ T cells in media containing CXCL12 neutralizing antibody for 24 h and then stained for p16^INK4A^ and GFP. The number of GFP positive cells was counted, and the results are presented as a bar graph. F and G) MC38 (control or mCXCL12 overexpressing) or CT26 (control or mCXCL12 overexpressing) mouse colon cancer cells were transplanted into 7 week old C57BL/6 or BALB/c mice, respectively. The mice were euthanized after 3 weeks, and the tumor size was analyzed. The tumor growth is presented as line graph. Results are presented as mean ± SD. Tumor tissues were stained with CXCL12 or CD8 antibodies. The arrow indicates the CD8^+^ cells. Mouse CD8 mRNA expression was analyzed and presented as dot graph. The infiltration of CD8^+^ T cells in tumors was analyzed (200×). Three randomly selected areas of the tumor tissue per animal were photographed and analyzed for CD8^+^ T cell infiltration; the results were averaged and then presented as a dot graph. In vitro cell proliferation assay. MC38 (control or mCXCL12 overexpressing) or CT26 (control or mCXCL12 overexpressing) cells were cultured, and the cell number was analyzed. Results are presented as mean ± SD. The *p* value was calculated by the Mann–Whitney *U* test (A, C, E, F, and G) or Kruskall–Wallis test (A, B, and D). *N* and *n* indicated the number of cases and independent experiments, respectively.

The physiologic concentration of CXCL12 elicits the chemotaxis of CD8^+^ T cells. However, it has been proposed that chemotactic movement is halted and even chemorepulsive movement can be triggered at above a certain level of CXCL12.^[^ ^27^ ^]^ As CXCL12 was expressed in senescent tumor cells, it was tempting to speculate that the CXCL12 secreted from senescent tumor cells might influence intratumoral CD8^+^ T cell infiltration. SW480 cells overexpressing CXCL12 were cocultured with isolated primary CD8^+^ T cells. T cell recruitment was limited in the CXCL12 overexpressing SW480 cells (**Figure** **3**A). Recombinant human CXCL12 (rhCXCL12) exerted a similar effect on CD8^+^ T cell migration. The migration of primary naïve and CD3/CD28 activated CD8^+^ T cells was induced at a low rhCXCL12 concentration (50 ng mL^−1^) and inhibited at high rhCXCL12 concentration (1 µg mL^−1^) (Figure 3B). Furthermore, when conditioned media (CM) from CXCL12 overexpressing cells were placed in the upper chamber, more Jurkat T cells migrated from the upper to the lower chamber (Figure 3C). Additionally, CXCL12 knockdown in ROS induced senescent tumor cells restored T cell attraction (Figure 3D). To exclude the indirect effect of senescent cells on CD8^+^ T cells, such as CXCL12‐dependent reduction of other T cell chemokine expression in SW480 cells by autocrine manner, we treated rhCXCL12 protein to SW480 cells and analyzed T cell chemokine expression. However, rhCXCL12 did not influence their expression (Figure S4, Supporting Information). Ex vivo culture data showed that the inhibition of CXCL12 by neutralizing antibodies in p16^INK4A^ positive tumors increased the infiltration of exogenously treated primary CD8^+^ T cells (Figure 3E). To analyze the effects of CXCL12 on CD8^+^ T cell infiltration using an in vivo animal model, we transplanted mouse CXCL12 (mCXCL12) overexpressing MC38 murine origin colon cancer cells into C57BL/6 mice. The tumor size was significantly larger in the mCXCL12 overexpressing MC38 cell‐transplanted group (Figure 3F), although in vitro cell growth rates were similar regardless of mCXCL12 expression. CD8^+^ T cell infiltration was markedly decreased in MC38 mCXCL12 transplanted tumors (Figure 3F). We further examined an in vivo animal model using mCXCL12 overexpressing CT26 mouse colon cancer cells transplanted in BALB/c mice, and the results were consistent with those of the MC38 experiment (Figure 3G).

### 2.4 High CXCL12 Concentrations Induce CXCR4 Loss in CD8^+^ T Cells

**Figure 4:**
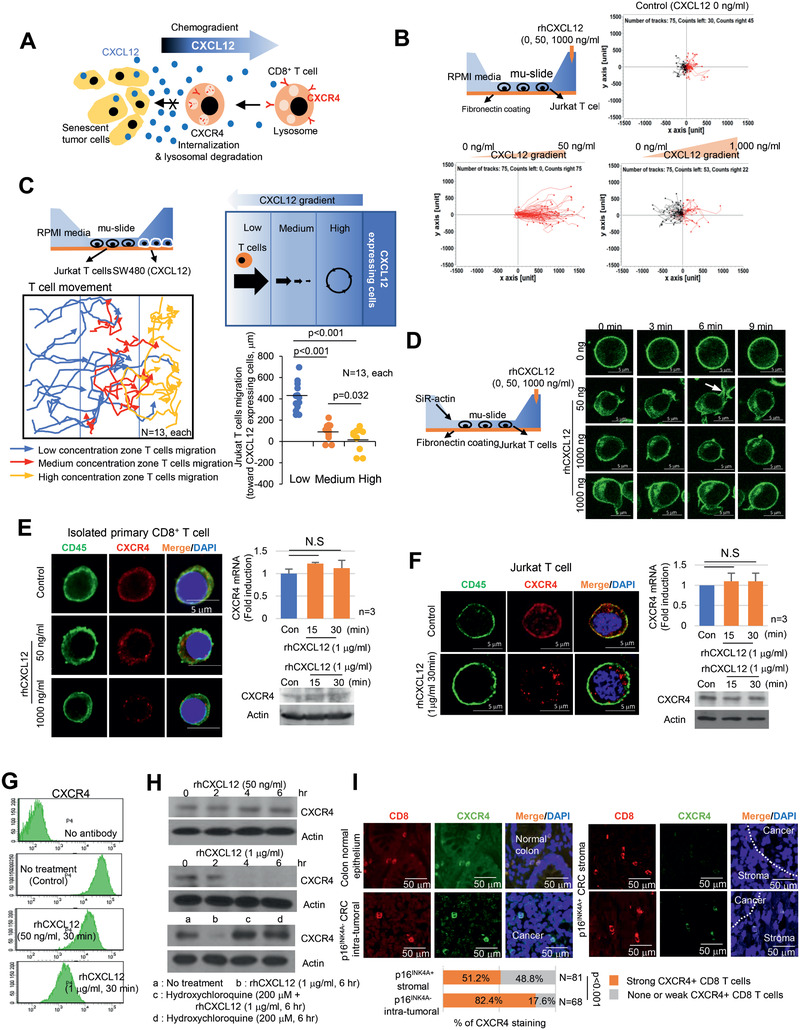
High concentrations of CXCL12 induce the loss of the plasma membrane CXCR4 in T cells. A) Schematic representation of CD8^+^ T cell migration in the presence of senescent tumor cells. B) CXCL12 chemogradient was developed using µ‐slide and then Jurkat T cell migration was analyzed. The migrated T cells were tracked, and the results are presented graphically (*n* = 75, each). The red and black lines indicate the tracks of the chemoattracted and chemorepulsed cells, respectively. C) The CXCL12 chemogradient was developed by CXCL12 overexpressing SW480 cells in µ‐slide, and T cell migration was analyzed. The migrated T cells were tracked, and the result is presented as a line graph (left panel). The migration distances were measured, and the result is presented graphically (lower right panel). The *p* value was calculated by one‐way ANOVA and post hoc analysis. D) Low and high concentrations of rhCXCL12 were treated with µ‐slide and lamellipodia formation was analyzed with SiR‐actin staining. The arrow indicates lamellipodia. E and F) CXCR4 expression in isolated primary CD8^+^ T cells E) and Jurkat T cells F). Isolated primary CD8^+^ T or Jurkat cells were treated with 50 or 1000 ng mL^−1^ of rhCXCL12 for 30 min and analyzed for CD45 by immunocytochemistry and CXCR4 expression by immunocytochemistry, real‐time PCR and western blot. G) Jurkat T cells were treated with rhCXCL12 (50 or 1000 ng mL^−1^) for 30 min and analyzed for CXCR4 expression in the plasma membrane by FACS. H) A high CXCL12 concentration induced CXCR4 lysosomal degradation. Jurkat T cells were treated with 50 or 1000 ng mL^−1^ rhCXCL12 for the indicated times and then analyzed for CXCR4 protein expression by western blotting. Jurkat T cells were treated with 1 µg mL^−1^ rhCXCL12 with or without 200 × 10^−6^ m hydroxychloroquine for 6 h and then analyzed for CXCR4 protein by western blotting. I) CXCR4 expression in CD8^+^ T cells in CRC. p16^INK4A^ positive and negative CRC tissues were immunostained for CD8 (red) and CXCR4 (green). The indicated cells were counted and presented as a bar graph (lower panel). The *p* value was calculated using the *χ* ^2^ test. *N* indicated the number of cases. NS indicates no significant.

We hypothesized that senescent tumor cells secrete high levels of CXCL12 and form a chemokine gradient from the tumor tissues to the stromal area. Therefore, CD8^+^ T cells located far from the senescent cells are attracted by low CXCL12 concentrations; however, as T cells approach the senescent cells, CXCR4 on CD8^+^ T cells is internalized and degraded. As a result, CD8^+^ T cells lose their directionality around the senescent tumor cells and thus cannot infiltrate the tumor tissues (**Figure** **4**A). To prove this hypothesis, we analyzed T cell migration using the mu‐gradient slide (µ‐slide).^[^ ^28^ ^]^ Expectedly, a low rhCXCL12 concentration (50 ng mL^−1^) attracted Jurkat T cells, indicating a chemoattractant effect. However, at high rhCXCL12 concentrations (1 µg mL^−1^) T cells lost their directionality, with some Jurkat T cells moving in the opposite direction (Figure 4B; Movie S1, Supporting Information). CXCL12 concentration dependent T cell chemotactic migration was also observed in the µ‐slide experiment using isolated human primary CD8^+^ T cells (Figure S5A, Supporting Information). Furthermore, Jurkat T cells migrated toward the CXCL12 expressing cells to some extent; however, when the cells reached a certain point, they lost their directionality. Jurkat T cells in the high concentration zone could not migrate toward but accumulated in front of the CXCL12 expressing cells (Figure 4C; Movie S2, Supporting Information). These chemotactic movements were specific to the CXCL12, since there was no chemotaxis at different concentration of CXCL10 in Jurkat T cells with lower CXCR3 expression levels compared to CXCR4 (Figure S5B, Supporting Information). We next analyzed actin polymerization in Jurkat T cells. At low CXCL12 concentrations, actin polymerization increased, forming lamellipodia in the direction of CXCL12; by contrast, at high CXCL12 concentrations, Jurkat T cells did not form lamellipodia (Figure S5C, Supporting Information). The CXCR4 inhibitor, AMD3100 treatment inhibited lamellipodia formation (Figure S5C, Supporting Information) and inhibited CD8^+^ T cell migration completely under low CXCL12 concentrations (Figure S5D, Supporting Information). In addition, AMD3100 is unable to restore lamellipodia formation and chemotactic migration at high CXCL12 concentrations. In the live imaging data, a low CXCL12 concentration induced lamellipodia formation toward the chemokine; however, under high CXCL12 concentrations, lamellipodia were irregularly shaped or did not form at all (Figure 4D; movie S3, Supporting Information). A study showed that CXCR4 could be internalized through ligand binding and was either recycled or subjected to lysosomal degradation. At high rhCXCL12 concentrations, the plasma membrane localized CXCR4 in Jurkat and primary CD8^+^ T cells was reduced within 30 min, although CXCR4 protein and mRNA expression remained unchanged (Figure 4E,F). The CXCR4 localized in the plasma membrane rapidly disappeared and was internalized into the cytoplasm, exhibiting a dot‐like appearance. Moreover, FACS analysis showed that a high rhCXCL12 concentration induced a marked loss of CXCR4 in the plasma membrane (Figure 4G). After removing rhCXCL12 as fresh media, the CXCR4 recovery rate in the plasma membrane was limited under a high concentration of rhCXCL12 (Figure S6, Supporting Information). Western blot analysis showed that CXCR4 protein expression was decreased in the presence of high rhCXCL12 concentrations. Furthermore, hydroxychloroquine inhibited CXCL12 induced receptor degradation (Figure 4H). These data suggest the internalization of CXCR4 and its subsequent lysosomal degradation rather than its recycling. Finally, we investigated CXCR4 protein expression in CD8^+^ T cells in vivo, and we found that its expression on CD8^+^ T cells was maintained in the infiltrated intratumoral region of p16^INK4A^ negative CRC, but it decreased or was absent in p16^INK4A^ positive CRC (Figure 4I). Furthermore, when we treated AMD3100 to the CXCL12 expressing MC38 tumor bearing mouse, CD8^+^ T cells did not infiltrate to the tumor tissue (Figure S7, Supporting Information). These data suggest that senescent tumor cells in CRC inhibit the intratumoral infiltration of CD8^+^ T cells through a high CXCL12 concentration induced loss of CXCR4 on CD8^+^ T cells that results in impaired directional migration.

### 2.5 Senescent Tumor Cells Polarize Monocytes to M2 Macrophages and Are Involved in CD8^+^ T Cell Inactivation

**Figure 5:**
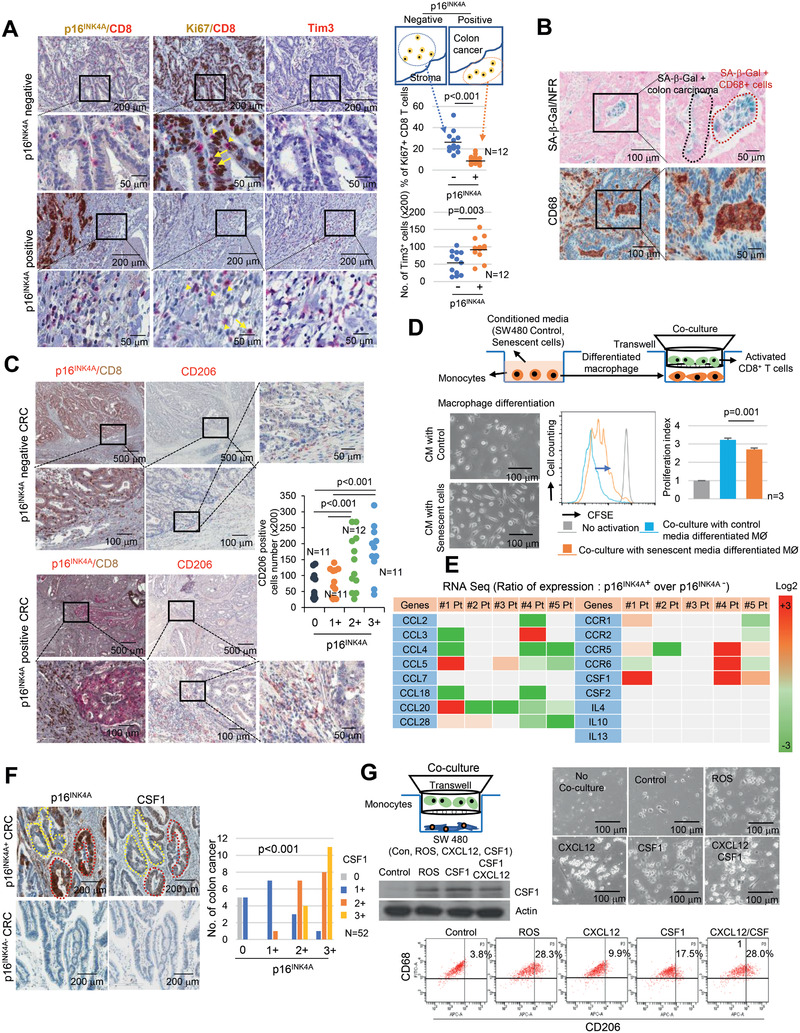
Senescent tumor cells inhibit CD8^+^ T cell activation via monocytes to M2 type macrophage differentiation. A) CD8^+^ T cells activation in p16^INK4A^ positive and negative CRC. p16^INK4A^ negative and positive CRC were dissected serially and immunostained with p16^INK4A^/CD8, Ki67/CD8 and Tim3, and the percentage of Ki67^+^/CD8^+^ cells was analyzed. Three randomly selected areas of the tumor tissue were photographed (200×) and then analyzed for Ki67^+^/CD8^+^ cell infiltration and Tim3^+^ cell number; the results were averaged and then presented as a dot graph. The *p* value was calculated by the Mann–Whitney *U* test. B) CD68 and SA‐*β*‐Gal staining analysis in CRC. C) Macrophage distribution analysis with CD206 in p16^INK4A^ positive or negative CRC. The number of CD206 positive macrophages in CRC according to the grades of p16^INK4A^ immunostaining was presented as a dot graph. D) T cell proliferation assay. Isolated primary monocytes were incubated with CM from SW480 or SW480/ROS for 7 days and then monocyte differentiation was analyzed (left lower panel). CFSE labeled CD8^+^ T activation by anti‐CD3/CD28 beads was performed under coculture with differentiated macrophages for 96 h, and suppression of T cell proliferation was measured by flow cytometry. The *p* value was calculated by one‐way ANOVA and post hoc analysis C,D). E) Microdissection analysis. The expression in RNA sequencing indicates the relative values of the p16^INK4A^ positive region compared with the p16^INK4A^ negative region. F) CRC tissues were serially sectioned for p16^INK4A^ and CSF1 immunostaining. The *p* value was calculated using the *χ* ^2^ test. G) CSF1 polarized monocytes to M2 macrophages. Isolated primary monocytes were cocultured with SW480 (control, H_2_O_2_ treated, CSF1 overexpressing, and CXCL12 overexpressing) for 6 days and then analyzed for cell morphology (upper panel), and CD206 expression by FACS (lower panel), respectively. *N* and *n* indicated the number of cases and independent experiments, respectively.

To determine whether the infiltrated CD8^+^ T cells were activated or exhausted, we performed CD8/Ki67 co‐immunostaining and found an increased number of activated CD8^+^ T cells in the intraepithelial of p16^INK4A^ negative CRC when compared to those cells in p16^INK4A^ positive CRC stromal (26.1% vs 9.9%). In addition, the number of Tim3 expressing cells increased in the stroma of p16^INK4A^ positive CRC (**Figure** **5**A). To clarify whether the senescent tumor cells inhibit T cell activation directly, we analyzed the rate of CD8^+^ T cell proliferation in the CM from the control (SW480) and from the senescent tumor cells (SW480/ROS). The proliferation indices of the T cells activated by CD3/CD28 did not significantly differ (Figure S8A, Supporting Information). Furthermore, CXCL12 overexpression did not show any effect on CD8^+^ T cell granzyme B levels as a proxy for T cell activation in the mouse model (Figure S8B, Supporting Information). These data suggest that neither senescent tumor cells themselves nor CXCL12 significantly influenced CD8^+^ T cell inactivation and that other factors were involved in CD8^+^ T cell exhaustion. In addition to CRC epithelial cells, we identified SA‐*β*‐Gal positive senescent cells being scattered in the stroma around the senescent tumor cells in Figure S1A (Supporting Information). These cells were strongly immune‐positive for CD68, indicating that they originated from monocytes; such cells include macrophages (Figure 5B). Recent studies have suggested that M2 macrophages express SA‐*β*‐Gal in response to SASP.^[^ ^29^, ^30^ ^]^ Therefore, we focused on macrophage differentiation. To examine the effects of senescent tumor cells on the macrophages, we performed immunohistochemical staining with CD163 and CD206 as markers for M2 macrophages and with HLA‐DR as a marker for M1 macrophages (Figure S8C, Supporting Information). The infiltration of CD206 positive cells in the stroma around the senescent tumor cells significantly increased compared to that around p16^INK4A^ negative tumor cells (Figure 5C). However, HLA‐DR expression was not significantly different (Figure S8D, Supporting Information). Furthermore, CD206 positive cells were found in the p16^INK4A^ positive senescent tumor cell region but were rarely identified in the p16^INK4A^ negative region of the same tumor (Figure S8E, Supporting Information). Furthermore, when we analyzed metastatic CRC in the liver, CD163^+^ macrophages accumulated in the primary and metastatic p16^INK4A^ positive regions (Figure S8F, Supporting Information). These data suggest that senescent tumor cells could be involved in the differentiation of monocytes into M2 type macrophages that results in CD8^+^ T cell inactivation. Therefore, T cell proliferation assay was performed by using CD8^+^ T cells cocultured with differentiated macrophage in the CM from control or senescent tumor cells (Figure 5D). The proliferation index of T cells was significantly decreased during coculture with differentiated macrophages by senescent tumor cells. These data suggest that senescent tumor cells inhibited CD8^+^ T cell activation via monocyte differentiation. Differentiation of monocytes into macrophages is regulated by several types of cytokines.^[^ ^31^ ^]^ Therefore, we compared the gene expression profiles (same scheme as in Figure 2C) through RNA sequencing. Among these molecules, we focused on the cytokines associated with macrophage differentiation, including interleukins (IL), CC chemokine ligands (CCLs), and colony stimulating factors (CSFs); we found that CSF1 was markedly upregulated in the senescent tumor cells of three out of five patients (Figure 5E). In contrast, IL4, IL10, IL13, and CSF2 were not expressed. Immunohistochemistry analysis showed that CSF1 expression was markedly increased in senescent tumor cells (Figure 5F). To explore the role of senescent tumor cells in macrophage polarization, we cocultured isolated primary monocytes with colon cancer cells (SW480), ROS induced senescent tumor cells (SW480/ROS), CXCL12, and/or CSF1 overexpressing cells. The isolated primary monocytes could be differentiated into M1 or M2 macrophages by lipopolysaccharide or IL4 treatment (Figure S9A, Supporting Information). Similar to ROS induced senescent tumor cells, CSF1 and CSF1/CXCL12 overexpressing cells induced the upregulation of CD206 and showed an increased expression of the mRNAs associated with M2 macrophages (Figure 5G; Figure S9B, Supporting Information). Furthermore, CM from senescent tumor cells induced the polarization of THP1 and U937 cells to M2 macrophage (Figure S9C, Supporting Information). CSF1 downregulation by shCSF1 in ROS induced senescent tumor cells decreased M2 macrophage differentiation (Figure S9D,E, Supporting Information). We further confirmed the M2 macrophage differentiation by using CSF1 overexpression or knockdown MC38 cells in mice. MC38 are high CSF1 expressing cells.^[^ ^32^ ^]^ Although tumor size was slightly decreased in the shCSF1‐infected tumor, knockdown of CSF1 by shCSF1 showed decreased infiltration of M2 macrophages in the tumors and was associated with an increase in the number of activated CD8^+^ T cells in the tumors (Figure S9F, Supporting Information). These data suggest that senescent tumor cells influence the polarization of macrophages and alter the tumor microenvironment, resulting in a cancer‐supportive environment that impairs the cytotoxic activity of CD8^+^ T cells.

### 2.6 CXCL12/CSF1 Inhibition Enhances ICI Efficacy

**Figure 6:**
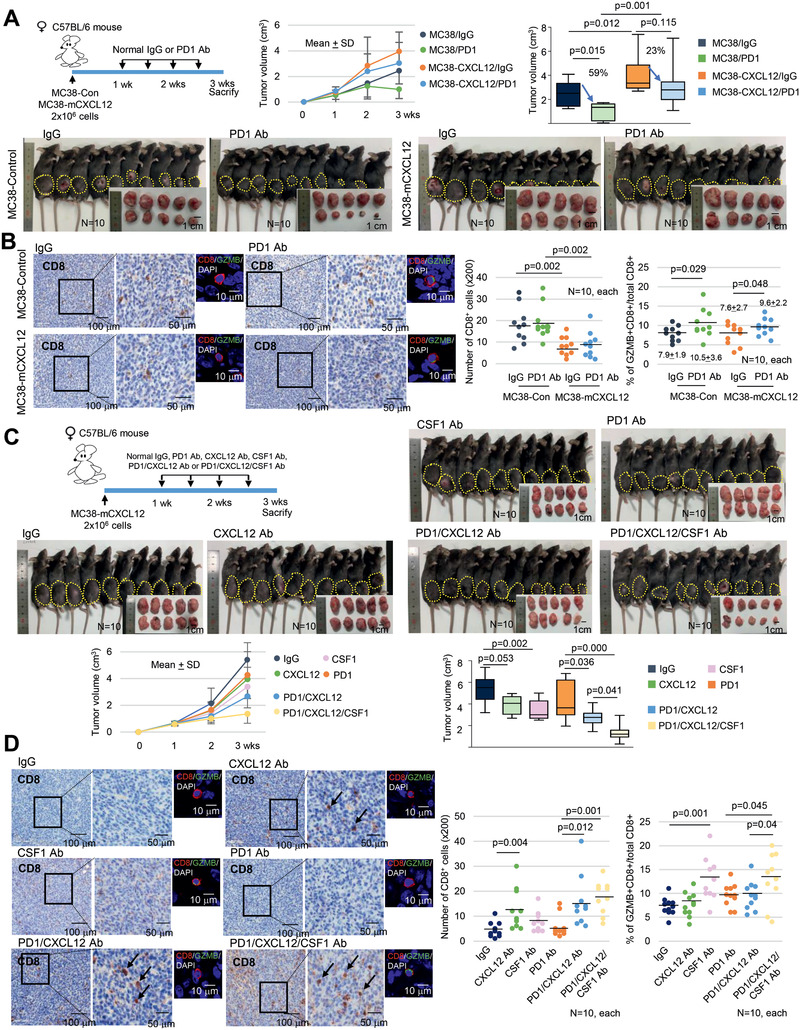
CXCL12 inhibits the ICI efficacy. A) MC38 cells (control, mCXCL12 overexpressing) were transplanted into C57BL/6 mice. One week later, the mice were treated with isotype control IgG or anti‐PD1 antibody through intraperitoneal injection twice per week for 2 weeks. The mice were euthanized, and the tumor volume was analyzed. B) CD8^+^ and Granzyme B (GZMB)^+^/CD8^+^ T cell infiltration was analyzed in MC38 derived tumor tissue in mice. C) mCXCL12 overexpressing MC38 cells were transplanted into C57BL/6 mice. One week later, the mice were treated with isotype control IgG or anti‐PD1 antibody with or without mCXCL12/mCSF1 neutralizing antibody through intraperitoneal injection twice per week for 2 weeks. The mice were euthanized, and the tumor volume was analyzed. D) CD8^+^ and GZMB^+^/CD8^+^ T cell infiltration was analyzed in MC38 derived tumor tissues from mice (200×). Tumor volume was presented as mean ± SD with line graph, and the final tumor volume was presented as a Box‐and‐Whisker diagram. The line inside the box is median. The top and the bottom of the box are the 75% and 25% percentile, respectively. Error bars on the whiskers represent minimum to maximum. In the case of T cells number, three randomly selected areas of the tumor tissue per animal were photographed and then analyzed for CD8^+^ and GZMB^+^/CD8^+^ cell infiltration; the results were averaged and presented as a dot graph. The *p* value was calculated using one‐way ANOVA and post hoc analysis A–D). *N* indicated the number of cases.

Based on our data, we hypothesized that senescent tumor cells build a cytokine shield around nonsenescent tumor cells that results in the inhibition of CD8^+^ T cell infiltration and in the regulation of monocyte differentiation. Disrupting this barrier through the elimination of senescent tumor cells can increase immunotherapeutic efficacy, as this action enhances the infiltration and activation of effector T cells. Unfortunately, a method that adequately eliminates senescent tumor cells is currently unavailable. Therefore, we combined the use of ICI with CXCL12/CSF1 targeting. First, we evaluated whether mCXCL12 overexpressing MC38 cells, which mimic the senescent tumor cells secreting CXCL12, could inhibit the effect of ICI by decreasing T cell infiltration. Similar to our previous experiment (Figure 3F), the tumor volumes in mCXCL12 overexpressing MC38 cells were significantly increased (*p* = 0.012, **Figure** **6**A), and the number of CD8^+^ T cells was significantly decreased compared with that of the control group (*p* = 0.002, Figure 6B). Anti‐PD1 antibody showed a significant therapeutic effect in control mice (*p* = 0.015), whereas, as expected, a limited effect was observed in mCXCL12 overexpressing MC38 transplanted mice (*p* = 0.115). Although the proportion of activated (granzyme B^+^) CD8^+^ T cells was increased after anti‐PD1 antibody treatment (Figure 6B), even without a significant change in total CD8^+^ T cell count in both groups, a decreased absolute number of CD8^+^ T cell in the intratumoral region of the mCXCL12 overexpressing MC38 tumor may be insufficient to show the antitumorigenic effect of ICI. Therefore, to overcome ICI resistance in this model, we subsequently applied mCXCL12 or mCSF1 neutralizing antibodies in mice transplanted with mCXCL12‐overexpressing MC38 cells that express a high basal level of mCSF1. After treatment with the mCXCL12 and/or mCSF1 neutralizing antibodies together with the anti‐PD1 antibody, although tumor growth was impaired not only with anti‐mCXCL12 but also with anti‐mCSF1 neutralizing antibody, the greatest decrease in tumor volume was exhibited by a combination of the anti‐mCSF1, anti‐mCXCL12, and anti‐PD1 treatment groups (Figure 6C). Intratumoral CD8^+^ T cell infiltration increased in the anti‐mCXCL12 antibody treated groups (Figure 6D). Furthermore, the percentage of activated CD8^+^ T cells was increased in the anti‐mCSF1 treated group (Figure 6D; Figure S10, Supporting Information). These data suggest that the elimination of CXCL12 and CSF1 can potentially improve immunotherapeutic efficacy through enhancement of CD8^+^ T cell infiltration and activation.

### 2.7 Inhibition of CXCL12 from Senescent Tumor Cells Suppress Tumor Progression in Azoxymethane (AOM)/Dextran Sulfate Sodium (DSS)‐Induced CRC Mouse Model

**Figure 7:**
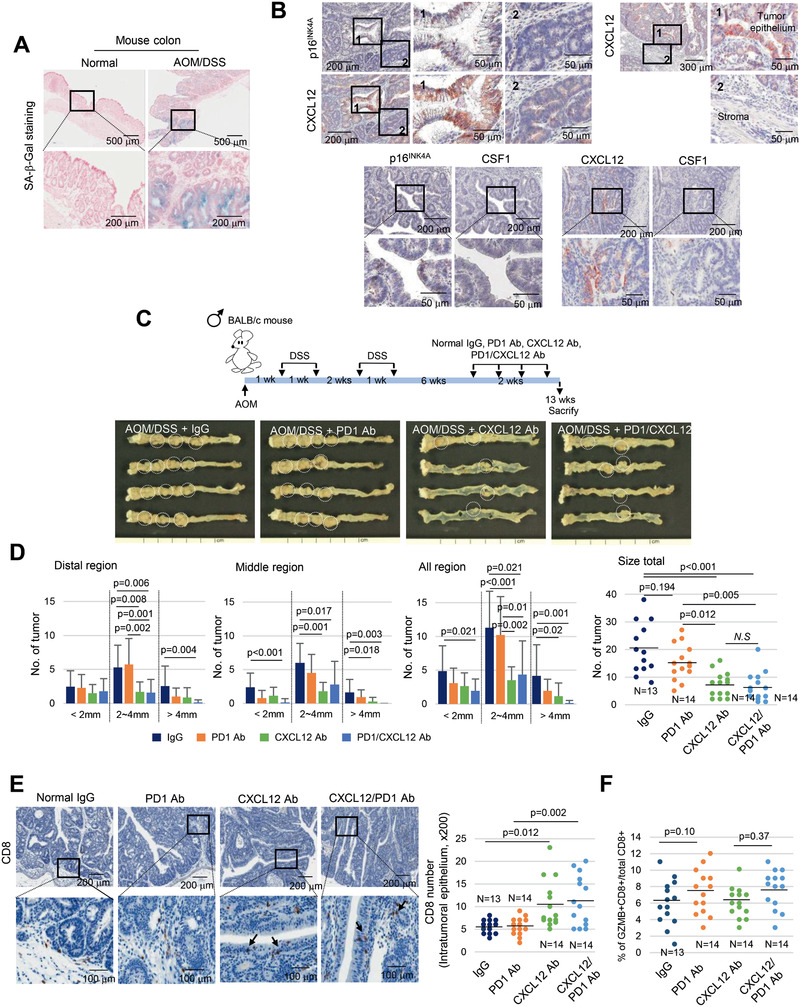
Neutralization of CXCL12 inhibits progression of AOM/DSS‐induced CRC. A) SA‐*β*‐Gal staining in normal mouse colon epithelium and AOM/DSS‐induced CRC. B) CXCL12 expression in p16^INK4A^ positive senescent tumor cells. AOM/DSS‐induced CRC tissues were serially dissected and stained with p16^INK4^, CXCL12 and CSF1 antibodies. “1” and “2” indicate the high‐magnification views of the original figure. C) Scheme of the AOM/DSS‐induced CRC and representative photograph of the colon tissues from each group. White circle indicates tumor nodules. D) Quantification of the number and size of tumors from each group. E) CD8^+^ T cell infiltration was analyzed in the AOM/DSS‐induced CRC tissues from each group (200×). Arrow indicates infiltrated CD8^+^ T cells. F) GZMB/CD8 double immunostaining was performed and the percentage of double positive cells were counted and presented as dot graph. Data are displayed as mean and the *p* value were calculated using one‐way ANOVA and post hoc analysis D–F).

To further validate the direct association between the presence of senescent tumor cells and the suppression of antitumor T cell immunity in CRC, we generated an AOM/DSS‐induced CRC mouse model that faithfully mimics human CRC.^[^ ^33^ ^]^ All the tumors contained SA‐*β*‐Gal and p16^INK4A^ positive senescent tumor cells (**Figure** **7**A,B). Similar to human CRC, increased CXCL12 expression was observed in p16^INK4A^ positive senescent mouse CRC. However, immunoexpression of CSF1 was not observed (Figure 7B). The expression of other T cell chemokines showed no statistically significant changes in AOM/DSS‐induced tumors, although the level of some chemokines (mCCL2 and mCXCL16) was increased (Figure S11, Supporting Information). CXCL12 expression was observed mainly in tumor epithelial cells but not in the stromal region (Figure 7B). Since p16^INK4A^ positive senescent tumor cells were identified in every tumor nodule, the correlation between senescent tumor cells and CD8^+^ T cell infiltration could not be analyzed as previously performed on human CRC. Nonetheless, since an increased frequency of CD8^+^ cytotoxic T cells can abrogate the AOM/DSS‐induced tumor growth,^[^ ^34^ ^]^ we wondered whether a reduction of CXCL12 levels from senescent tumor cells can augment the effect of ICI or inhibit tumor growth directly. In our AOM/DSS‐induced model, the group treated with anti‐PD1 antibody showed a slight decrease in tumor frequency and size without statistical significance, similar to the results of a previous study.^[^ ^35^ ^]^ However, surprisingly, the frequency and size of tumors was significantly reduced in the groups treated with anti‐CXCL12 antibody, regardless of anti‐PD1 antibody treatment (Figure 7C,D). Additionally, we found increased CD8^+^ T cell infiltration of the intratumoral epithelium in the groups treated with anti‐CXCL12 antibody as compared with those of the control group (Figure 7E). The frequency of activated CD8^+^ T cells did not differ regardless of anti‐PD1 and/or anti‐CXCL12 antibody treatments (Figure 7F). Consequently, these data from the AOM/DSS‐induced CRC model further support that senescent tumor cells build a cytokine barrier around nonsenescent tumor cells, protecting them from attack by the CD8^+^ cytotoxic T cells through inhibition of their effective infiltration.

## 3 Discussion

Recent multicenter studies have demonstrated that the densities of CD3^+^ and CD8^+^ T cells are the most important markers for predicting the prognosis of CRC.^[^ ^14^, ^15^ ^]^ Therefore enhancing endogenous antitumor T cell responses in CRC by ICI therapy could be an important clinical issue. In CRC, however, the durable benefits of ICI therapy are limited to a minority of cases with MSI.^[^ ^36^ ^]^ Furthermore, the mechanisms involved in the resistance to ICI therapy in the majority of the MSS CRC cases remain unclear. In the present study, we are the first to report that the presence of senescent tumor cells in MSS CRC was significantly associated with a low density of intratumoral CD8^+^ T cell infiltration. Senescent tumor cells can generate a chemical barrier and create a supportive tumor microenvironment. We found that among the SASP, CXCL12/CSF1 played a pivotal role in the exclusion and inactivation of CD8^+^ T cells, which are the most important policeman‐like cells in our body.

Although CXCL12 was initially identified as a chemoattractant for CD8^+^ T cells,^[^ ^37^ ^]^ Feig et al. suggested that CXCL12, which is secreted by carcinoma‐associated fibroblasts, could induce T cell exclusion in pancreatic cancer.^[^ ^38^ ^]^ However, in our CRC cohort, immunohistochemical analyses revealed that CXCL12 is abundantly expressed in senescent tumor cells but not in stromal fibroblasts; CXCL12 expressed in CDX2 positive CRC cells but not in vimentin positive cells (Figure S12A–C, Supporting Information), findings that are consistent with a previous study.^[^ ^39^ ^]^ When we directly compared the concentration of CXCL12 in senescent tumor cells and in adjacent stromal cells individually isolated by microdissection, the CXCL12 concentration in the senescent tumor cells was significantly higher than that in adjacent stromal cells (Figure S12D, Supporting Information). Recent studies have suggested that CXCL12 exerts different chemoattractant effects depending upon its concentration.^[^ ^27^, ^40^, ^41^ ^]^ In the present study, a high CXCL12 concentration induced the loss of polarity of CD8^+^ T cells through the loss of the CXCR4 receptor in the plasma membrane. When an adequate amount of ligand binds to the receptor, CXCL12 transmits signals downstream and is involved in cell migration and survival. However, the ligand is abundant in the extracellular space, and the receptor is internalized into the cytoplasm and undergoes lysosomal degradation. At this point, the in vivo CXCL12 concentration that can sufficiently induce the loss of directional movement remains unknown. To titrate the CXCL12 chemokine activity more precisely, we treated a rhCXCL12 dose dependently and found that less than 250 ng mL^−1^ of rhCXCL12 exhibits T cells attractive activity, but over this concentration results in loss of CD8^+^ T cell polarity (Figure S5D, Supporting Information) and CXCL12 overexpressing cells showed as high as 300 ng mL^−1^ (Figure 3A). Although CXCL12 concentrations in CXCL12 overexpressing cancer cells and senescent tumor cells in the cancer tissue were not directly compared, the distance between the zone of polarity loss and CXCL12‐expressing cells in the µ‐slide experiment (approximately 200 µm) was similar to that between p16^INK4A^ and CD8^+^ T cells (Figure S12E, Supporting Information).

In addition to CXCL12 overexpression, senescent tumor cells can inhibit the infiltration of CD8^+^ T cells into tumor tissues through insufficient production of T cell‐attracting chemokines, physical barriers, or aberrant vasculature.^[^ ^42^, ^43^, ^44^ ^]^ CXCL9, CXCL10, and CXCL11 have a major role in attracting tumor‐specific T cells into cancers,^[^ ^45^, ^46^ ^]^ and the insufficient expression of these chemokines at the tumor favors immune evasion.^[^ ^47^ ^]^ In this study, the levels of CXCL9 and CXCL11 were insufficient, and CXCL10 was further decreased in senescent tumor cells. Furthermore, since stromal cells, including immune cells and vimentin positive fibroblasts, also expressed CXCR4 (Figures S3D and S13A, Supporting Information), it is possible that fibroblasts, a major cell type in the composition of the extracellular matrix, is involved in immune cell infiltration. CXCL12 secreted from senescent tumor cells could stimulate fibroblasts by paracrine effect and the activated fibroblast can change the microenvironment around cancer cells by secretion of other chemokines. However, such changes were not observed in the CRC or in vitro experiments (Figures S3A and S13B, Supporting Information).

M2 macrophage can suppress CD8^+^ T cell activity via M2 macrophage‐derived cytokines and proteases.^[^ ^48^ ^]^ For examples, TGF*β* inhibits antitumor activity of CD8^+^ T cell by suppressing the expression of several cytolytic genes, including granzyme A, granzyme B, IFN‐*γ*, and FAS ligand.^[^ ^49^ ^]^ Furthermore, M2 macrophage‐derived arginase 1 causes dysregulation of the T cell receptor signal and subsequently induces CD8^+^ T cell unresponsiveness.^[^ ^50^ ^]^ M2 macrophage differentiation can be induced by various cytokines but is most prominently accomplished by IL4 and IL13.^[^ ^31^ ^]^ In addition, CSF1, CCL2, and CCL5, a major chemokine for monocyte recruitment, can also induce M2 macrophage differentiation.^[^ ^51^ ^]^ Our RNA sequencing data showed the IL4 and IL13 were not expressed in CRCs. Although CCL2 expression in the entire cancer tissues was slightly increased overall compared with that of the normal tissues, CCL2 and CCL5 expression were not related to p16^INK4A^ positive tumor cells (Figure S14, Supporting Information). However, the expression of CSF1 was significantly higher in p16^INK4A^ positive tumor cells. Although previous studies have identified fibroblasts as a main source of inflammatory cytokines, including CSF1, CSF1 expression was observed both in cancer epithelial cells and in vimentin positive cells. Moreover, the extent and intensity of CSF1 immunostaining and of the ELISA data showed that the senescent tumor cells were the major sources of CSF1 (Figure S12A,B, Supporting Information). Therefore, based on our data, we suggest that senescent tumor cells are major cells that induce M2 macrophage polarization that can inhibit CD8^+^ T cell activity.

To date, we do not know the exact mechanisms through which senescent tumor cells in invasive CRC tissues are developed. Cellular senescence can be induced by persistent DNA damage signaling, which can be caused by various stimulations (oncogene activation, chemotherapy, ROS, etc.). Endogenous ROS has been proven to promote tumor migration and invasion.^[^ ^52^ ^]^ In our current study, p16^INK4A^ senescent cells were more frequently observed on the invasive margin of CRC and were correlated with lymph node metastasis. Tumor cells at the invasive front of CRC have been found to be associated with hypoxia,^[^ ^24^ ^]^ which stimulates the production of mitochondrial ROS by reoxygenation.^[^ ^25^ ^]^ On the basis of these findings, we speculate that the main inducer of senescence in CRC is possibly ROS production. Hif1*α* has been known to be expressed in the hypoxia region, and ROS was generated with loss of Hif1*α* expression during reoxygenation.^[^ ^53^ ^]^ Interestingly, Hif1*α* was expressed opposite to p16^INK4A^ expression in CRC (Figure S15, Supporting Information). Moreover, the profile of the secretory cytokines of the ROS induced senescent colon cancer cell was similar to that of the in vivo p16^INK4A^ senescent cells of CRC tissue. However, further studies are required to gain an understanding of the exact mechanism of cellular senescence in tumor tissues.

Nonetheless, since ROS did not induce cellular senescence but rather induce apoptosis in MC38 (p53 mutant) and CT26 (homozygous deletion of Cdkn2a) cells, in vivo CRC model which can mimic the presence of p16^INK4A^ senescent cells in human CRCs was required. However, since the exact mechanism of tumor cell senescence was not discovered, it was difficult to determine which model would be closest to the current phenotype. AOM/DSS‐induced mouse CRC contained SA‐*β*‐Gal and p16^INK4A^ positive senescent tumor cells like those seen in human CRC and the p16^INK4A^ positive senescent tumor cells secreted CXCL12 abundantly. However, unlike human CRC, the senescent tumor cells did not secrete CSF1 in AOM/DSS‐induced mouse CRC. Potential binding sites of the transcription factors, including NF*κ*B, Myc, and STAT1/2, are present in human and mouse CSF1 promoter. However, other predictive binding sites for p53, CEBPB, and the Hox family, only exist in the human CSF1 promoter. Therefore, some SASP including CXCL12^[^ ^54^, ^55^ ^]^ can be expressed by activation of common transcription factor such as NF*κ*B in both species, however, other SASP including CSF1 can be heterogeneously regulated by the difference of promoter sequences. In addition to species‐to‐species differences, there are several studies showing that SASP is quite heterogenous even within the same cell or tissue, depending on the senescence inducers.^[^ ^56^, ^57^, ^58^ ^]^

Therefore, considering the heterogeneity of SASP, it may be more potent to eliminate senescent tumor cells themselves than to block the function of SASP. However, in this study, we could not apply the methods that target senescent tumor cells because the methods or drugs that selectively kill senescent tumor cells have not been extensively developed. Although several senolytic drugs, such as dasatinib and quercetin, have been developed^[^ ^59^, ^60^ ^]^ to selectively target senescent fibroblasts and spare normal cells, these drugs do not seem to effect on senescent tumor cells. The results of our preliminary experiment revealed that these drugs did not induce apoptosis in senescent tumor cells. Therefore, targeting SASP is considered to be a more effective approach. In this study, we surprisingly found a dramatic reduction in tumor sizes in mice treated with CXCL12/CSF1 neutralizing antibodies together with anti‐PD1 antibody, and CD8^+^ T cell infiltration was markedly increased. Likewise, in AOM/DSS‐induced mouse CRC containing senescent tumor cells, CXCL12 neutralizing antibody induced robust CD8^+^ T cell infiltration into the intratumoral epithelium along with a significant reduction in the number and size of tumors. These findings suggest that inhibiting the specific secretome of senescent tumor cells can amplify the effect of ICI by eliminating the T cell‐exclusive cytokine shield.

**Figure 8:**
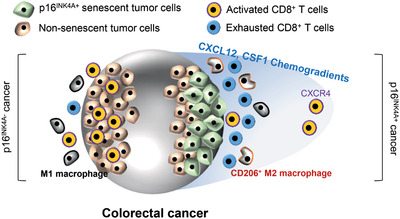
Schematic representation of the role of senescent tumor cells in CRC on CD8^+^ T cell infiltration and macrophage differentiation.

In the tumor microenvironment, the role of senescent tumor cells remains to be elucidated. We previously reported that senescent tumor cells play a role in collective invasion.^[^ ^9^ ^]^ In this study, we suggest another significant role in cancer progression through immune suppression. In the battle between cancer and immune cells, senescent tumor cells stand at the forefront of combat and form a protective barrier, thereby inhibiting CD8^+^ T cell infiltration and activation. Senescent tumor cells express a variety of SASP to establish a shield surrounding the cancer cells and build a supportive tumor microenvironment (**Figure** **8**). Therefore, eliminating senescent tumor cells or targeting SASP is proposed as a new strategy for inhibiting cancer progression through blocking the adverse effects of senescent tumor cells and enhancing the efficacy of cancer immunotherapy.

## 4 Experimental Section

##### Colorectal Cancer Samples from Patients

CRC samples were obtained from patients with informed consent at Ajou University Hospital after surgical resection. Fresh tumor and normal tissues for SA‐*β*‐Gal staining were separately sampled in the representative areas by an experienced pathologist immediately after resection and divided into two identical tissue sections. One was used to snap frozen in liquid nitrogen immediately and other was processed for FFPE, according to the tissue specimen regulation of Ajou University Hospital. Patients who had a past history of chemotherapy or radiation therapy before the surgery were excluded from the study.

##### Histopathological Analysis

Review of CRC tissue sections was performed independently by an experienced pathologist (JHK). The immune infiltration in CRC was accessed with modification of methods previously described by the International Immune‐Oncology Biomarkers Working Group^[^ ^61^ ^]^ and by Pagès et al.^[^ ^62^ ^]^ Briefly, the fields analyzed were chosen as representative of the CRC region in which showed most highly infiltrated by immune cells and were at distance from necrotic material or abscesses. A percentage of area occupied by mononuclear inflammatory cells over total intratumoral/tumor marginal area was analyzed with magnification 200×. All mononuclear cells (including lymphocytes and plasma cells) were included, but polymorphonuclear leukocytes (neutrophils) were excluded. Next, the immune infiltration was graded as weak (less than 10%), moderate (10–40%), or strong (more than 40%).

##### Primary Monocytes and CD8^+^ T Cells Isolation and Activation

Monocytes and CD8^+^ T cells were isolated from human peripheral blood mononuclear cells (PBMCs), which were obtained from the Korean Redcross Blood Services with the approval of the Institutional Review Board of Ajou University Hospital (AJIRB‐BMR‐SMP‐17‐424). The PBMCs were isolated using Ficoll Paque Plus (GE Healthcare Life Sciences, Buckinghamshire, UK). The monocytes were isolated using a pan monocyte isolation kit (MACS 130‐096‐537, Miltenyi Biotech, Gladbach, Germany), whereas the CD8^+^ T cells were isolated using a CD8^+^ T cell isolation kit (MACS, 130‐096‐495, Miltenyi Biotech). The monocytes and CD8^+^ T cells were maintained in complete Roswell Park Memorial Institute (RPMI) medium with 10% fetal bovine serum (FBS, GIBCO‐BRL, Grand Island, NY). To obtain activated T cells, the isolated primary CD8^+^ T cells with Dynabeads Human T‐Activator CD3/CD28 (Thermo Fisher Scientific, Inc., Waltham, MA) was incubated for 72 h and were used for migration assay.

##### T Cell Proliferation Assay

The isolated primary CD8^+^ T cells were labeled with 5 × 10^−6^ m carboxyfluorescein diacetate succinimidyl ester (CFSE, Invitrogen, Carlsbad, CA) and incubated for 15 min at 37 °C in the dark. Following incubation, fluorescence was quenched by further incubation in a serum‐containing medium. The cells were stimulated with Dynabeads Human T‐Activator CD3/CD28 for 72 or 96 h under the indicated culture condition. Proliferating CD8^+^ T cells were tracked by flow cytometry. The percentages of T cells within each cell division were identified through subgating for each division and the proliferation index was calculated as previously described.^[^ ^63^ ^]^

##### Cancer Cell Lines

SW480, U937, THP‐1, Jurkat T (clone E6‐1), and CT26 cells were purchase from Korean Cell Line Bank (KCLB, Seoul, Korea). SW480, U937, THP‐1, and Jurkat T cells were maintained in complete RPMI media with 10% FBS and CT26 was maintained in complete Dulbecco's Modified Eagle's Medium (DMEM) media with 10% FBS. MC38 cell was purchase from Kerafast (Boston, MA) and it was maintained in complete DMEM media with 10% FBS. ROS induced senescent tumor cells were generated by H_2_O_2_ (200 × 10^−6^ m) treatment for 4 days.

##### Animal Preparation

MC38‐control cells (1 × 10^6^), MC38‐mouse CXCL12 cells (MC38‐mCXCL12, 1 × 10^6^), MC38‐mouse CSF1 cells (MC38‐mCSF1, 1 × 10^6^), MC38‐mouse shCSF1 cells (MC38‐shmCSF1, 1 × 10^6^), CT26‐control cells (1 × 10^6^) or CT26‐mouse CXCL12 cells (1 × 10^6^) were resuspended in 100 µL of phosphate‐buffered saline (PBS) and implanted subcutaneously into female or male C57BL/6 or BALB/c mouse, respectively (7 weeks old) and then sacrificed the mice 3 weeks later. To inhibit CXCR4 signaling, AMD3100 (3 mg kg^−1^) were intraperitoneally injected once a day for 2 weeks after one week after subcutaneous injection of MC38‐mCXCL12 cells (2×10^6^). In the case of immune check point inhibitor treatment experiments, after implanted the tumor cells (2 × 10^6^ cells), one week later, normal isotype immunoglobulin G (mouse IgG2a; clone C1.18.4, rat IgG2a; clone 2A3, rat IgG1k; clone HRPN, BioXcell, West Lebanon, NH) or anti‐PD‐1 antibody (10 mg kg^−1^, BioXcell, clone RMP1‐14) were intraperitoneally injected twice a week for 2 weeks. In the case of the CXCL12 and CSF1 inhibition study, the animal model scheme was modified.^[^ ^64^, ^65^ ^]^ MC38‐mCXCL12 cells (2 × 10^6^) were resuspended in 100 µL of PBS and implanted subcutaneously into female C57BL/6 mice (7 week old). One week later, normal isotype immunoglobulin G (Control‐IgG) or anti‐PD1 antibody (10 mg kg^−1^) with or without anti‐CXCL12 (500 µg kg^−1^, Merck, Darmstadt, Germany, clone K15C) or anti‐CSF1 (7.5 mg kg^−1^, BioXcell, clone 5A1) neutralizing antibody was intraperitoneally injected twice a week for 2 weeks. Tumor volume calculations were obtained using the formula *V* = (width^2^ × length)/2 for caliper measurements.

##### AOM/DSS‐Induced CRC Mouse Model

BALB/c mice (6 weeks old; *N* = 56) were intraperitoneally injected with 10 mg kg^−1^ AOM (A5486, Sigma, St. Louis, MO) followed by two cycles of 2% DSS (160110, MP Biomedical, Solon, OH) for 7 days in weeks 2 and 5. Except for one mouse that died after the second cycle of DSS treatment, the remaining mice were divided into four treatment groups (isotype control IgG:13, anti‐PD1 antibody:14, anti‐CXCL12 antibody:14, anti‐PD1/CXCL12 antibody:14) according to body weight. Normal isotype IgG (mouse IgG2a; clone C1.18.4, rat IgG2a; clone 2A3), anti‐PD1 antibody (10 mg kg^−1^, BioXcell, clone RMP1‐14), and anti‐CXCL12 (500 µg kg^−1^, Merck, clone K15C) neutralizing antibodies were intraperitoneally injected twice a week from week 11 to 12. The mice were weighed weekly and sacrificed at week 13. The entire colon from the anus to cecum was dissected longitudinally and cleaned with PBS to examine tumor nodules. The colon was divided by one‐third and designated as proximal, middle, and distal, respectively.

##### Immunohistochemistry and Immunocytochemistry

Immunohistochemistry was performed by the Benchmark XT automated processor (Ventana Medical Systems Inc, Tucson, AZ) on 4 µm thick representative tissue sections of formalin fixed paraffin‐embedded tissues. The primary antibodies used were as follows: p16^INK4A^, predilution (805‐4713, Roche, Tucson, AZ); p16^INK4A^, 1:100 (ab54210, Abcam, Cambridge, MA); CXCR4, 1:100 (MAB172, R&D System, Minneapolis, MN); CXCL9, 1:100 (ab9720, Abcam); CXCL10, 1:100 (MAB2662, R&D System); CXCL11, 1:100 (ab9955, Abcam); CXCL12, 1:100 (MAB360, R&D System); CXCL16, 1:100 (GTX632502, GeneTex); CD45, 1:100 (LCA 2B11&PD7/26, Cell Marque, Rocklin, CA); CD8, 1 µg mL^−1^ (NBP2‐29475, Novus Biologicals, Littleton, CO); CD8, 1:400 (#98941, Cell Signaling Technology, Danvers, MA); FoxP3, 5 µg mL^−1^ (MAB8214, R&D System); CD3, 1:100 (MRQ‐39, Cell Marque); CD4, predilution (790‐4423, Ventana Medical Systems Inc); CD206, 8 µg mL^−1^ (MAB25341, R&D System); HLA‐DR, 0.1 µg mL^−1^ (ab20181, Abcam); CD68, 1:100 (IR609, Dako Denmark); CD163, 1:50 (MRQ‐26, Cell Marque); Ki67, 1:100 (MIB‐1, Dako); CDX2, 1:100 (EPR2764Y, Cell Marque); CSF1, 1:100 (PA5‐42558, Invitrogen, Carlsbad, CA); Histone 3 K9 tri‐methylation, 1:100 (EPR16601, Abcam); Tim3, 1:500 (ab241332, Abcam); Granzyme B (1:100, ab4059, Abcam; 1:200, 14‐8822‐82, Invitrogen); Hif1*α*, 1:100 (NB100‐479, Novus Biologicals); CCL2, 1:100 (MAB2791, R&D System); CCL5, 1:100 (MAB278, R&D System). Detection was done using the Ventana Optiview DAB kit (Ventana Medical Systems). Double immunohistochemistry was performed with the UltraView universal DAB detection kit (#760‐500, Ventana Medical Systems Inc) for first antibodies and then with the UltraView Universal Alkaline Phosphatase Red Detection kit (#760‐501, Ventana Medical Systems Inc.) for second antibodies in the Benchmark XT automated immunohistochemistry stainer. For the immunocytochemical staining was performed with primary antibodies; CD45, 1:200 (ab8216, Abcam); CXCR4, 1:100 (ab28842, Abcam); F‐actin 1:500 (P1951, Sigma). Slides were washed two times with PBS and incubated with appropriate conjugated secondary antibodies for 1 h at room temperature. Secondary antibodies for immunocytochemistry were as follows: Alexa Fluor 488, 1:600 (A‐21206, Thermo Fisher Scientific, Waltham, MA); Alexa Fluor 555, 1:600 (A‐31572, Thermo Fisher Scientific). In the case of F‐actin staining, slides were applied with rhodamine phalloidin for 1 h and then analyzed with fluorescence microscope. Immunostaining was scored as positive if the cytoplasm or nucleus showed a moderate or strong intensity of staining and was scored negative if none or a weak cytoplasmic or nuclear staining was present. Immunostainings for p16^INK4A^, CXCL12, and CSF1 were grades according to the proportion of immunopositive cells (0: less than 1%; 1+: 1–20%; 2+: 20–40%; and 3+: more than 40% of cancer cells).

##### Ex Vivo Culture

Cancer tissues were isolated from p16^INK4A^‐positive and negative CRC. Collected cancer tissues were washed with PBS three times and incubated at 37 °C for 24 h with GFP tagged primary isolated CD8^+^ T cells in completed RPMI medium. In the case of CXCL12 neutralizing antibody treatment, 5 µg mL^−1^ of anti‐CXCL12 antibody (R&D System) was applied to the medium for 24 h. The tissues were fixed with formalin for 24 h and perform immunohistochemistry with p16^INK4A^, CD8 and GFP antibodies.

##### RNA Sequencing Analysis

Total RNA was extracted from p16^INK4A^ positive and negative region after LMD7 laser microdissection (Leica, Wetzlar, Germany) using Macherey‐Nagel RNA kit (Macherey‐Nagel GmbH & Co. KG, Düren, Germany). Briefly, the sample quality was checked using Bioanalyzer RNA Chip (Agilent Technologies) and RNA sequencing running was carried out with Nextseq 500 (Illumina, San Diego, CA). RNA sequencing data were deposited in GEO (GSE125253).

##### Senescence Associated *β*‐Galactosidase (SA‐*β*‐Gal) Staining

The frozen tissue slides were fixed with 10% formalin (Sigma) for 1 min and then incubated with SA‐*β*‐Gal solution (X‐gal, 1 mg mL^−1^; citric acid/sodium phosphate, pH 5.8, 40 × 10^−3^ m; potassium ferrocyanide, 5 × 10^−3^ m; potassium ferricyanide, 5 × 10^−3^ m; NaCl, 150 × 10^−3^ m; MgCl_2_, 2 × 10^−3^ m) for 12 h at 37 °C. The cultured cells were fixed with 4% paraformaldehyde (Sigma) and incubated with SA‐*β*‐Gal solution. After washing with PBS, SA‐*β*‐Gal‐positive cells were then analyzed under light microscopy.

##### Migration Assay

Migration of the cells was assessed by Transwell (5 µm pore size, 24 well, Corning, NY). CD8^+^ T cells, Jurkat, or monocytes were place in upper chamber which was filled with 100 µL of serum free RPMI. Cells or CXCL12 contained serum free RPMI or complete RPMI were place in the lower chamber as a chemoattractant. Migrated cells were counted as suspension cells in lower chamber.

##### Immunoblotting

Cells were lysed in RIPA buffer (Tris pH 7.5, 20 × 10^−3^ m; NaCl, 150 × 10^−3^ m; 1% Nonidet P‐40; 0.5% sodium deoxycholate; EDTA, 1 × 10^−3^ m; 0.1% SDS) containing protease inhibitor cocktail (K272, Biovision, Milpitas, CA) and phosphatase inhibitor cocktail (K282, Biovision). Samples were then resolved by SDS‐PAGE and immunoblotted with the indicated antibodies; CXCR4, 1:500 (ab28842, Abcam); CSF1, 1:1000 (PA5‐42558, Invitrogen); actin, 1:3000 (Abc‐2004, Abclon, Seoul, Korea).

##### Cloning of Human and Mouse CXCL12, CSF1, and Lentivirus Preparation

cDNAs of human and mouse CXCL12, human CSF1, and mouse CSF1 were cloned from normal human fibroblasts and mouse fibroblasts in the laboratory. cDNAs were inserted into the pCDH‐CMV‐MCS‐EF1‐Puro lentivirus vector (System Biosciences, Mountain View, CA). To generate lentiviral particles, HEK‐293TN cells were transfected with plasmid DNA (pGagpol, pVSV‐G, and pCDH‐human CXCL12, pCDH‐mouse CXCL12, pCDH‐human CSF1, pCDH‐mouse CSF1). For knockdown of CSF1 and CXCL12 expression, shRNA was prepared in a pLKO lentiviral vector (Sigma) and then amplified in 293TN cells. Cells were plated and grown in 6 cm culture dishes. After overnight culture, they were infected with lentivirus and then the cells were selected with 4 × 10^−6^ m puromycin for 1 week. shRNA sequences were as follows: sh‐CSF1 #1: 5′‐TCTCCTGGTACAAGACATAATCTC‐3′; sh‐CSF1 #2: 5′‐AGATCCAGTGTGCTACCTTAACTC‐3′ sh‐CXCL12 #1: 5′‐ACATCTCAAAATTCTCAACACA‐3′; sh‐CXCL12 #2: 5′‐CGCCAACGTCAAGCATCTCAAA‐3′, sh‐mCSF1 #1: 5′‐GATAGACCATGCGCTTTAAACTC‐3′ sh‐mCSF1 #2: 5′‐ GCCTACCAAGACTGGATGAAACTC‐3′, respectively.

##### Real‐Time PCR Analysis

First‐strand cDNA was synthesized by SuperScript III First‐Strand Synthesis System (Invitrogen) from 1 µg of total cellular RNA. Real‐time PCR was carried out with Power SYBR Green PCR Master Mix (Bio‐Rad, Hercules, CA) using the following conditions: initial activation at 95 °C for 5 min, followed by 40 cycles of 95 °C for 15 s and 60 °C for 1 min. The primers used for real‐time PCR were given as follows: CXCL12: 5′‐TGCCAGAGCCAACGTCA‐3′, 5′‐CAGCCGGGCTACAATCTGAA‐3′; CXCR4: 5′‐GCCTTATCCTGCCTGGTATTGTC‐3′, 5′‐GCGAAGAAAGCCAGGATGAGGAT‐3′; CSF1: 5′‐CCAGGAACAGTTGAAAGATCCA‐3′, 5′‐TTATCTCTGAAGCGCATGGTGT‐3′; mouse CSF1: 5′‐AGTATTGCCAAGGAGGTGTCAG‐3′, 5′‐ ATCTGGCATGAAGTCTCCATTT‐3′; CSF2: 5′‐GGCCAGCCACTACAAGCAGCACT‐3′, 5′‐CAAAGGGGATGACAAGCAGAAG‐3′; CCL20: 5′‐ATGTGCTGTACCAAGAGTTT‐3′, 5′‐CAAGTCTGTTTTGGATTTGC‐3′; IL‐6: 5′‐AAGCCAGAGCYGTGCAGATGAGTA‐3′, 5′‐TGTCCTGCAGCCACTGGTTC‐3′; MMP‐1: 5′‐AAGCCAGAGCTGTGCAGATGAGTA‐3′, 5′‐TGTCCTGCAGCCACTGGTTC‐3′; MMP‐3: 5′‐CGCCTGTCTGAAGATGATATAAAT‐3′, 5′‐CTGACAGCATCAAAGGACAA‐3′; IL‐12p35: 5′‐GATGGCCCTGTGCCTTAGTA‐3′, 5′‐TCAAGGGAGGATTTTTGTGG‐3′; CXCL11: 5′‐CCTGGGGTAAAAGCAGTGAA‐3′, 5′‐TGGGATTTAGGCATCGTTGT‐3′; CCR7: 5′‐GTGGTGGCTCTCCTTGTCAT‐3′, 5′‐TGTGGTGTTGTCTCCGATGT‐3′; TGF*β*1: 5′‐TGCGCTTGAGATCTTCAAA‐3′, 5′‐GGGCTAGTCGCACAGACCT‐3′; MRC1(CD206): 5′‐GGCGGTGACCTCACAAGTAT‐3′, 5′‐ACGAAGCCATTTGGTAAACG‐3′; SR‐B1: 5′‐TGTGGGTGAGATCATGTGG‐3′, 5′‐GTTCCACTTGTCCACGAGGT‐3′; CCL2: 5′‐AGCAGCAAGTGTCCCAAAGA‐3′, 5′‐TTGGGTTTGCTTGTCCAGGT‐3′; CCL4: 5′‐GCTGCTCAGAGACAGGAAGT‐3′, 5′‐ACAGGAACTGCGGAGAGGAG‐3′; CCL5: 5′‐TCCCACAGGTACCATGAAGGTC‐3′, 5′‐GCAATGTAGGCAAAGCAGCAG‐3′; CXCL9: 5′‐TGCAAGGAACCCCAGTAGTGA‐3′, 5′‐GGTGGATAGTCCCTTGGTTGG‐3′; CXCL16: 5′‐CCTATGTGCTCTGCAAGAGGAG‐3′, 5′‐CTGGGCAACATAGAGTCCGTC‐3′; mouse CD8 5′‐CAGAGACCAGAAGATTGTCG‐3′, 5′‐TGATCAAGGACAGCAGAAGG‐3′; mouse granzyme B: 5′‐ACTCTTGACGCTGGGACCTA‐3′, 5′‐AGTGGGGCTTGACTTCATGT‐3′; mouse perforin: 5′‐GATGTGAACCCTAGGCCAGA‐3′, 5′‐GGTTTTTGTACCAGGCGAGA‐3′; mouse CD127: 5′‐TTTCTGCCCAATGATCTTCC‐3′, 5′‐CAGGGGACCTAGAGGAAAGG‐3′; mouse CCL2: 5′‐AAAAACCTGGATCGGAACCAA‐3′, 5′‐CGGGTCAACTTCACATTCAAAG‐3′; mouse CCL3: 5′‐ CACCCTCTGTCACCTGCTCAA‐3′, 5′‐ATGGCGCTGAGAAGACTTGGT‐3′; mouse CCL4: 5′‐CCAGGGTTCTCAGCACCAA‐3′, 5′‐GCTCACTGGGGTTAGCACAGA‐3′; mouse CCL5: 5′‐ACACCACTCCCTGCTGCTTT‐3′, 5′‐GACTGCAAGATTGGAGCACTTG‐3′; mouse CXCL9: 5′‐TCTGCCATGAAGTCCGCTG‐3′, 5′‐CAGGAGCATCGTGCATTCCT‐3′; mouse CXCL10: 5′‐TGCTGGGTCTGAGTGGGACT‐3′, 5′‐CCCTATGGCCCTCATTCTCAC‐3′; mouse CXCL11: 5′‐CGGGATGAAAGCCGTCAA‐3′, 5′‐TATGAGGCGAGTCTTTATGCTGGCAAACCTGCTTGCTTGG‐3′; mouse CXCL12: 5′‐AAGGCTGACATCCGTGGGAGAT‐3′, 5′‐GTCTTTATGCTGGCAAACCT‐3′; mouse CXCL16: 5′‐CAACCCTGGGAGATGACCAC‐3′, 5′‐CTGTGTCGCTCTCCTGTTGC‐3′; mouse *β*‐actin: 5′‐TGTCCACCTTCCAGCAGATGT‐3′, 5′‐AGCTCAGTAACAGTCCGCCTAGA‐3′; 18s: 5′‐CGGCTACCACATCCAAGGAA‐3′, 5′‐GCTGGAATTACCGCGGCT‐3′; *β*‐actin: 5′‐CCCTGGCACCCAGCAC‐3′, 5′‐GCCGATCCACACGGAGTAC‐3′, respectively.

##### ELISA Analysis

Cells (3 × 10^5^) were seeded in 24‐well plates and incubated for 48 h, after which the media were harvested. CXCL12 or CSF1 secretion into the culture media was measured using CXCL12 (DSA00, R&D Systems) or CSF1 ELISA kits (RayBiotech Life, Peachtree Corners, GA) according to the manufacturer's instructions. In the case of measurement of CXCL12 in the CRC, CRC tissues were serially dissected and stained with SA‐*β*‐Gal or toluidine blue. SA‐*β*‐Gal positive tumor epithelial regions and adjacent stromal regions (more than 30 paired regions/each case, *N* = 4) were microdissected and then dissolved in PBS and subsequently measured the CXCL12 concentrations using ELISA.

##### FACS Analysis

Jurkat T cells harvested and washed two times with PBS, cells were mixed with mouse antihuman CXCR4‐PE‐CY5 (15‐9999‐42, 1:40, Invitrogen). The corresponding mouse immunoglobulin (Ig) G2a‐PE‐CY5 isotype was used as a negative control. Cocultured monocytes were dissociated with 0.05% trypsin to a single cell suspension and incubated with mouse antihuman CD68‐FITC (562117, 1:40, BD Biosciences, San Jose, CA), mouse antihuman CD206‐APC (550889, 1:40, BD Biosciences). After 30 min incubation in the dark at room temperature, transferred to 5 mL polystyrene round bottom tubes and subjected to flow cytometry (BD FACSCanto II; BD Biosciences) for acquisition and analysis.

##### Monocytes Differentiation

Isolated primary monocytes were cocultured with control, ROS treated, CXCL12 overexpressed or CSF1 overexpressed SW480 cells using Transwell (0.4 µm pore size, 6 well, Corning) or cultured with conditioned media from control (SW480) or senescent tumor cells (ROS induced senescent SW480). After 6 day incubation removed upper chamber and monocytes were dissociated to a single cell and performed FACS and real time PCR analysis.

##### Chemotaxis Assays and Migration Track Analysis

The migration of cells under different chemokine gradients was analyzed using ibiTreat 2D “mu (µ)‐Slide” Chemotaxis system (Ibidi GmbH, Martinsried, Germany).^[^ ^28^ ^]^ Before the chemotaxis assay, the surface of the slide for was coated with fibronectin (F2006, 300 µg mL^−1^, Sigma) for 1 h for cells attachment. After washing with PBS, 6 µL of cells (3 × 10^6^ cells mL^−1^) were loaded into the central observation channel and incubated at 37 °C for 45 min to allow attachment. Gradients of CXCL12 were generated according to the manufacturer's instructions. For the migration track analysis, live cell imaging was recorded for indicated periods and intervals on a JuLi stage system (NanoEnTek, Seoul, Korea) operating on a CO_2_ incubator. The tracking of migrating individual cells was achieved using the Manual Tracking plugin (Institut Curie, Orsay, France) in ImageJ software (NIH, Bethesda, MA). Chemotaxis plots were obtained with the Chemotaxis and Migration plugin from Ibidi. To analyze the actin dynamics of live cells during chemotaxis, the cells were stained with SiR‐Actin, a fluorogenic, cell permeable and highly specific probe for F‐actin (SC001, 1 × 10^−6^ m, Spirochrome, Stein am Rhein, Switzerland) for 1 h before seeding onto “mu‐Slide” and time‐lapse images were obtained using confocal microscope (Nikon) with a 60× oil objective and a temperature‐controlled chamber (37 °C, 5% CO_2_).

##### Microscope Image Acquisition

For image acquisition of histology and immunohistochemical staining, ScanScope CS system (Aperio Technologies Inc., Vista, CA) was used at room temperature. The cell images were acquired using an Olympus microscope mounted with an Olympus DP70 digital camera and DP‐Manager software (Olympus Microscope Corp., Tokyo, Japan). Immunofluorescence images were collected on a Zeiss LSM 510 microscope and analyzed with Zeiss Axio Imager software (Carl Zeiss, Jena, Germany).

##### Statistical Analysis

Numerical data are presented as mean ± SD of independent determinations. Statistical analysis of differences was performed by the Mann–Whitney *U* test, Kruskall Wallis test, or one‐way ANOVA and post hoc analysis and a *p* value < 0.05 was considered as significant. To analyze the correlation between grades of p16^INK4A^ immunostaining and clinicopathologic parameters, *χ* ^2^‐test for trend was used when appropriate. Analyses were done by using The IBM SPSS software ver. 22.0 (IBM, Armonk, NY).

##### Study Approval

Obtaining CRC samples from patients with informed consent after surgical resection was approved by the Institutional Review Board of Ajou University Hospital (AJIRB‐BMR‐OBS‐16‐218). All animal experiments were approved by the institutional animal research ethics committee at Ajou University Medical Center (approval number: 2017‐0049).

## Conflict of Interest

The authors declare no conflict of interest.

## Author Contributions

Y.W.C. and Y.H.K. contributed equally to this work. Y.W.C, Y.H.K., J.H.K., H.S.K., and T.J.P. designed and contributed to analysis and interpretation of data. ; Y.W.C., Y.H.K., Y.J.P., Y.S.K., G.Y.L., J.E.Y., S.S.P., Y.K.L., and T.J.P. maintained cell and experiments including animal experiment. S.Y.O., K.W.S., S.Y.P., and J.H.K. provided patients’ samples and performed immunohistochemistry. Y.W.C. J.H.K., and T.J.P. wrote the manuscript.

## Supporting information

Supporting Information

Supplemental Movie 1

Supplemental Movie 2

Supplemental Movie 3
