# Supplementary material for: Senescent Tumor Cells Build a Cytokine Shield in Colorectal Cancer
Source: Adv Sci (Weinh). 2021 Jan 4;8(4):2002497. doi: 10.1002/advs.202002497 (PMC7887594; doi:10.1002/advs.202002497)
Supplement: Supplementary file 1 — Supporting Information [file ADVS-8-2002497-s001.pdf]

© 2021 Wiley-VCH GmbH

ADVANCED SCIENCE
Open Access

Supporting Information

for *Adv. Sci.,* DOI: 10.1002/advs.202002497

Senescent Tumor Cells Build a Cytokine Shield in Colorectal Cancer

Yong Won Choi, Young Hwa Kim, Seung Yeop Oh, Kwang Wook Suh, Young-Sam Kim, Ga-Yeon Lee, Jung Eun Yoon, Soon Sang Park, Young-Kyoung Lee, Yoo Jung Park, Hong Seok Kim, So Hyun Park, Jang-Hee Kim, and Tae Jun Park**

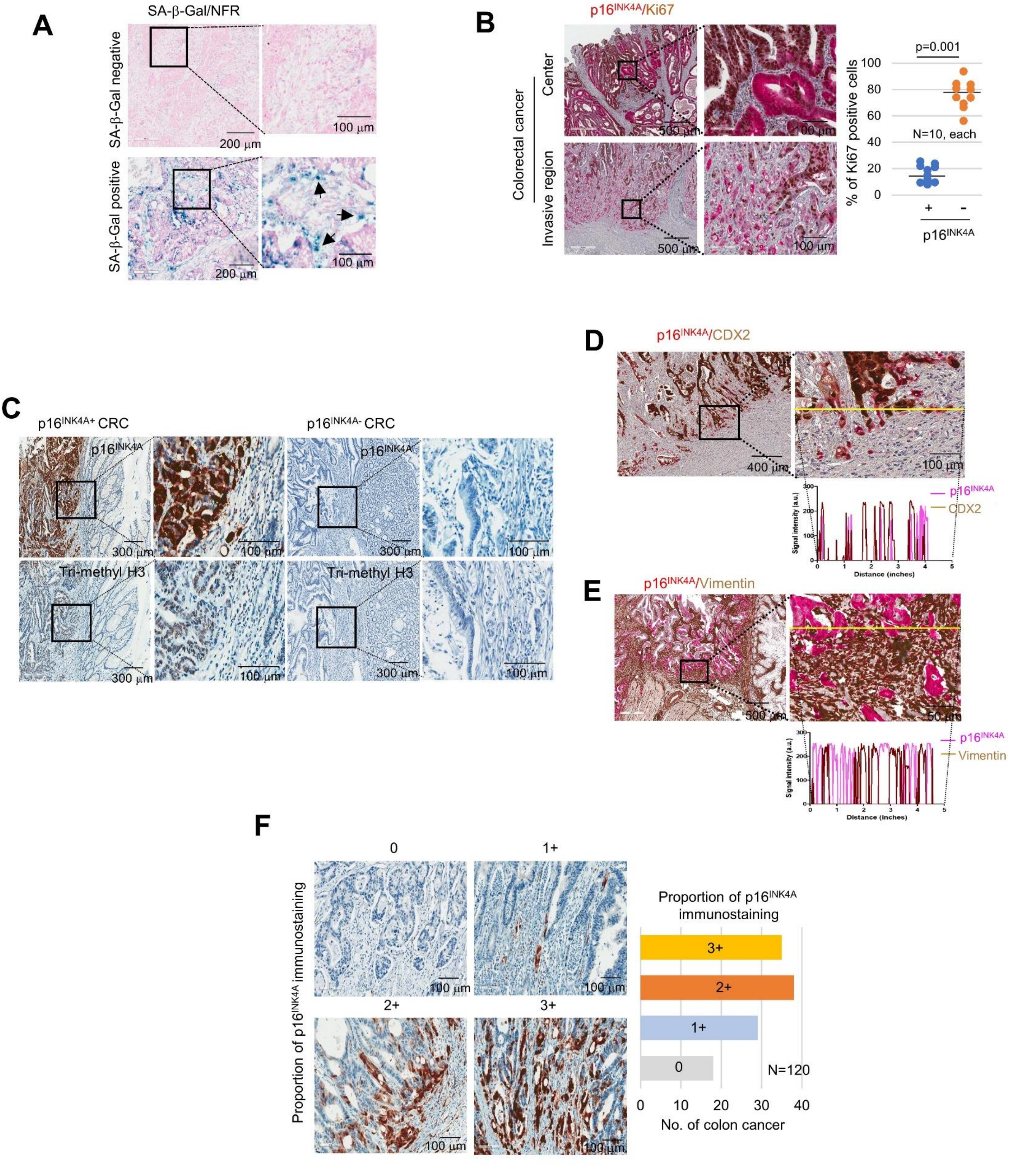
A
SA-β-Gal/NFR
SA-β-Gal negative
SA-β-Gal positive
200 µm
100 µm
B
p16INK4A/Ki67
Colorectal cancer
Center
Invasive region
500 µm
100 µm
p=0.001
% of Ki67 positive cells
N=10, each
+
-
p16INK4A
C
p16INK4A+ CRC
p16INK4A- CRC
p16INK4A
Tri-methyl H3
300 µm
100 µm
D
p16INK4A/CDX2
400 µm
100 µm
Signal intensity (a.u.)
Distance (inches)
p16INK4A
CDX2
E
p16INK4A/Vimentin
500 µm
50 µm
Vimentin
F
0
1+
2+
3+
Proportion of p16INK4A immunostaining
100 µm
N=120
No. of colon cancer

Figure S1. Characterization of SA-β-Gal-positive tumor cells and adjacent stromal cells. **A**) SA-β-Gal-positive round-shaped cells (arrow) in the stromal region of CRC frozen tissues that were stained for SA-β-Gal/NFR. The upper and lower panels show the SA-β-Gal-negative and SA-β-Gal-positive areas, respectively. **B**) CRC tissues stained with p16^{INK4A} (red)/Ki67 (brown). Ki67 positivity (proliferation activity) was analyzed in p16^{INK4A} positive and negative CRC. **C**) p16^{INK4A} positive and negative CRC tissues dissected serially and immunostained with p16^{INK4A} and histone 3 K9 trimethyl (Trimethyl H3), respectively. **D, E**) CRC tissues stained with p16^{INK4A} (red)/CDX2 (brown) (D) or p16^{INK4A} (red)/vimentin (brown) (E). The red and brown color were extracted and the intensity of each splitted image was measured by Image J software. The values were presented as peak graphs. **F**) One hundred and twenty cases of MSS CRC were graded as 0, 1+, 2+, or 3+ according to the proportion of p16^{INK4A}. The *p* value was calculated by Mann–Whitney U test (B). N indicated the number of cases.

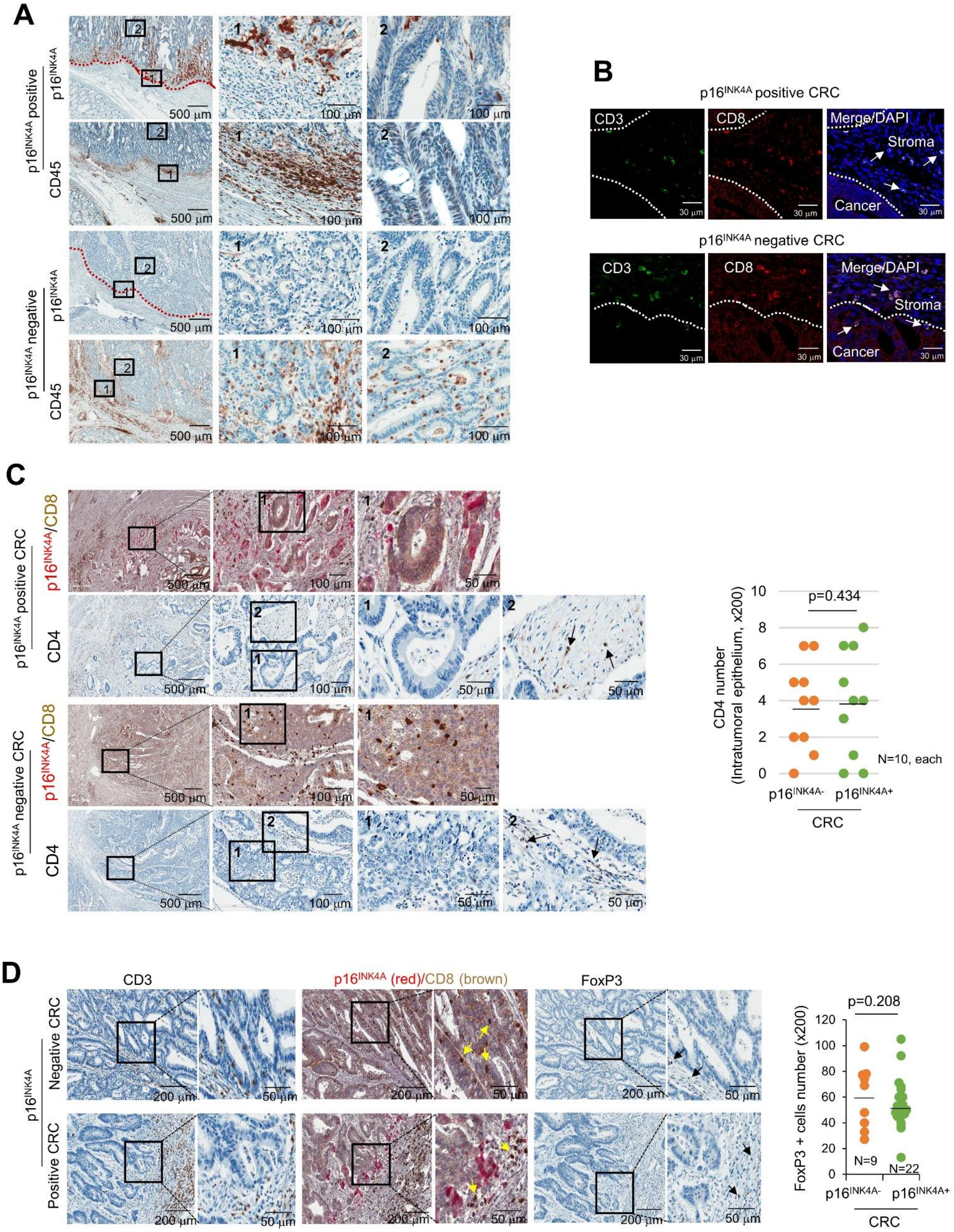

A
p16INK4A positive
p16INK4A
CD45
p16INK4A negative
p16INK4A
CD45
500 μm
100 μm
B
p16INK4A positive CRC
CD3
CD8
Merge/DAPI
Stroma
Cancer
30 μm
p16INK4A negative CRC
C
p16INK4A positive CRC
p16INK4A/CD8
CD4
p16INK4A negative CRC
50 μm
p=0.434
CD4 number
(Intratumoral epithelium, x200)
N=10, each
p16INK4A-
p16INK4A+
CRC
D
CD3
p16INK4A (red)/CD8 (brown)
FoxP3
p16INK4A
Negative CRC
Positive CRC
200 μm
p=0.208
FoxP3 + cells number (x200)
N=9
N=22

Figure S2. Characterization of CRC stromal cells. A) Infiltration of CD45 positive cells in $p16^{INK4A}$ positive and negative CRC. The center and the right columns show highly magnified views of the areas denoted as 1 and 2, respectively. The dotted line indicates the cancer margin. **B**) $p16^{INK4A}$ positive and negative CRC tissues were double immunofluorescence stained with CD3 and CD8. White arrows indicate CD3/CD8 double stained cells. **C**) $p16^{INK4A}$ positive and negative CRC tissues were dissected serially and stained with $p16^{INK4A}$/CD8 or CD4, respectively. Arrows indicate CD4 positive cells. Intratumoral infiltrated $CD4^+$ T cell numbers were analyzed and presented as dot graph. **D**) $p16^{INK4A}$ positive and negative CRC tissues were stained with CD3, $p16^{INK4A}$ (red)/ CD8 (brown), and FoxP3, and the number of FoxP3 positive cells was counted (x200). Yellow and black arrows indicate CD8 and FoxP3 positive cells, respectively. The *p* value was calculated by Mann–Whitney U test (C, D). N indicated the number of cases.

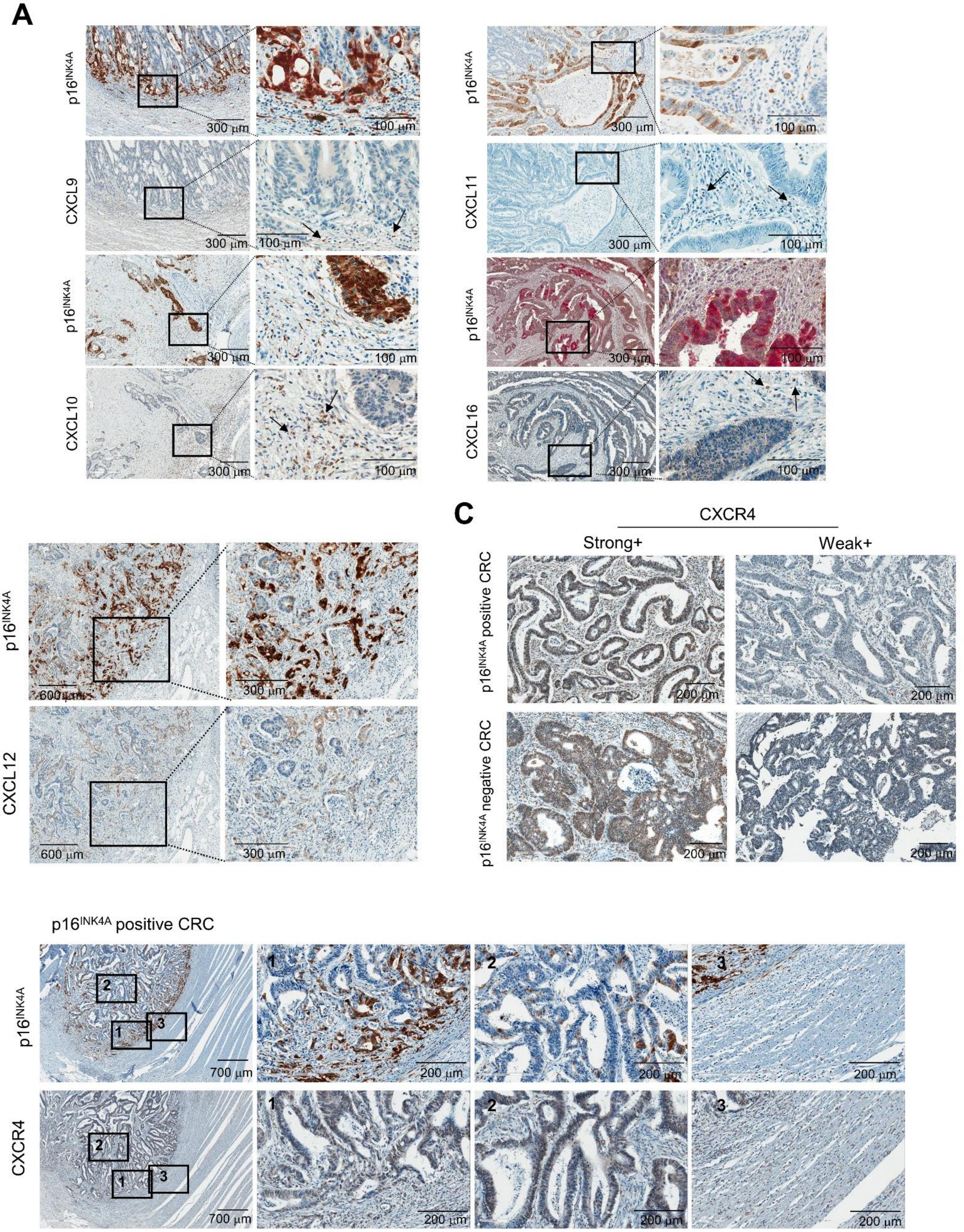

A
p16INK4A
CXCL9
p16INK4A
CXCL10
p16INK4A
CXCL11
p16INK4A
CXCL16
300 µm
100 µm
B
p16INK4A
CXCL12
600 µm
300 µm
C
CXCR4
Strong+
Weak+
p16INK4A positive CRC
p16INK4A negative CRC
200 µm
D
p16INK4A positive CRC
p16INK4A
CXCR4
1
2
3
700 µm
200 µm

Figure S3. T cell Chemokine and CXCR4 expression in CRC. A) Immunostaining for $p16^{INK4A}$, CXCL9, CXCL10, CXCL11, and CXCL16. $p16^{INK4A}$ positive CRC tissues were serially dissected and immunostained for $p16^{INK4A}$ CXCL9, CXCL10, CXCL11, and CXCL16. The arrows indicate the CXCL9, CXCL10, CXCL11, and CXCL16 positive cells. **B**) CXCL12 expression in $p16^{INK4A}$ expressing senescent tumor cells. $p16^{INK4A}$ positive CRC tissues were serially dissected and stained with $p16^{INK4A}$ and CXCL12 antibody, respectively. **C**) $p16^{INK4A}$ positive and negative CRC tissues were stained with CXCR4 antibody and presented as strongly- and weakly-CXCR4 positive CRC. **D**) CXCR4 expression in $p16^{INK4A}$ expressing senescent tumor cells. $p16^{INK4A}$ positive CRC tissues were serially dissected and stained with $p16^{INK4A}$ and CXCR4 antibodies. "1", "2", and "3" indicate the high magnification views of the original figure.

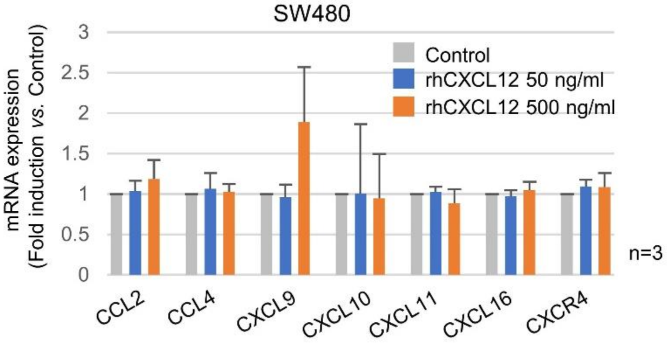

Figure S4. T cell chemokine expression in SW480 cells after treatment of CXCL12. SW480 cells were treated with a low concentration (50 ng/ml) or a high concentration (500 ng/ml) of rhCXCL12 for 24 hours and then analyzed CXCL9, CXCL10, CXCL11, CXCL16, CCL2, CCL4, and CXCR4 mRNA expression by real time PCR. CCL3 and CCL5 was not detected.

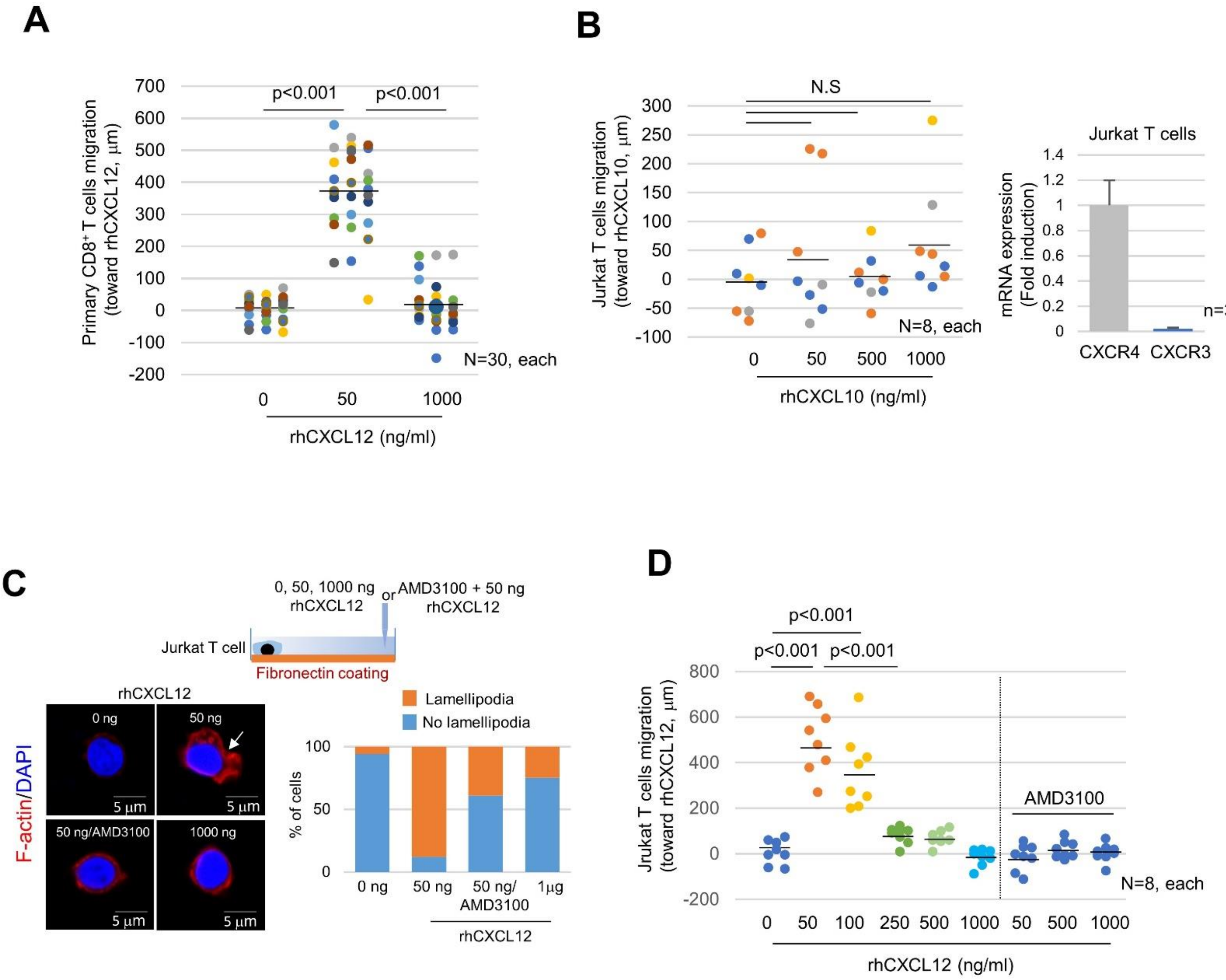

Figure S5. Chemotaxis of primary CD8$^+$ T and Jurkat T cells. A) CXCL12 chemogradient was developed using µ-slide, and then isolated primary CD8$^+$ T cell migration was analyzed. The migrated T cells were tracked, and the migration distances were measured. The result is presented graphically. **B**) CXCL10 chemogradient was developed using µ-slide, and then Jurkat T cell migration was analyzed. The migrated T cells were tracked, and the migration distances were measured. The result is presented graphically. CXCR4 and CXCR3 mRNA expression in Jurkat T cell was analyzed by real time PCR. **C**) Low (50 ng/ml) or high (1000

ng/ml) concentrations of rhCXCL12 were treated for 10 minutes and then analyzed for lamellipodia formation through F-actin staining. One hundred cells were counted and presented as a bar graph. The arrow indicates the lamellipodia. **D**) Titration of CXCL12. CXCL12 chemogradient was developed using μ-slide and then Jurkat T cell migration was analyzed. In the case of AMD3100 treated groups, rhCXCL12 (50, 500, 1000 ng/ml) and AMD3100 (5 μM) were cotreated and then T cells migration was measured. The migrated T cells were tracked, and the migration distances were measured, and the result is presented graphically.

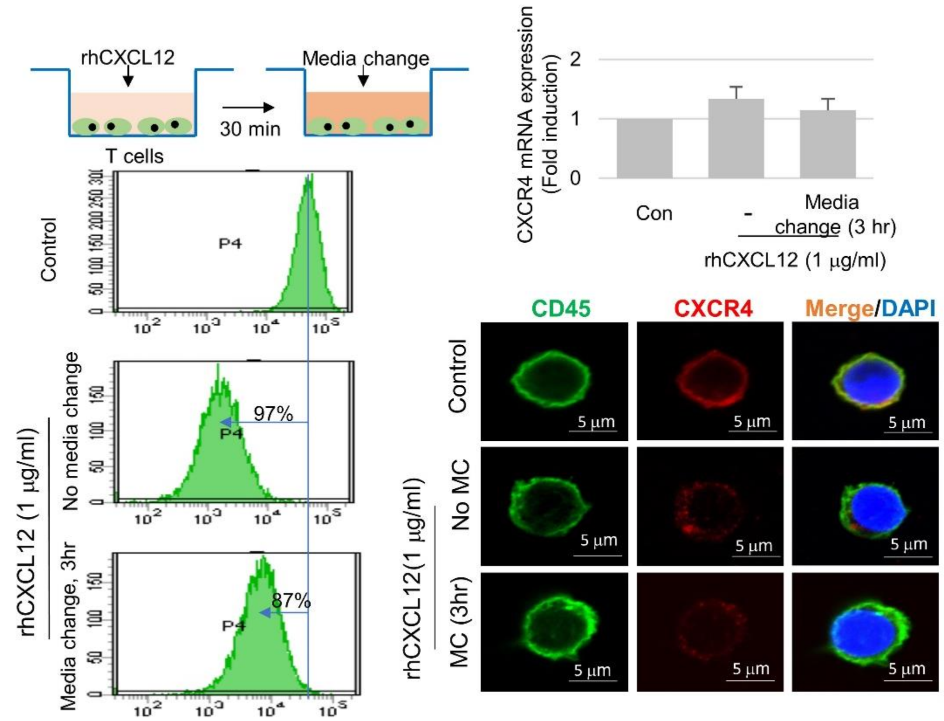

Figure S6. Recovery rate of CXCR4 in the plasma membrane under high rhCXCL12 concentrations. Jurkat T cells were treated with 1 μg/ml rhCXCL12 for 30 minutes. The cells were washed and added to fresh media and maintained for 3 hours. CXCR4 expression was analyzed by FACS (left panel), mRNA (upper right panel), and immunocytochemistry (lower right panel). MC indicates media change.

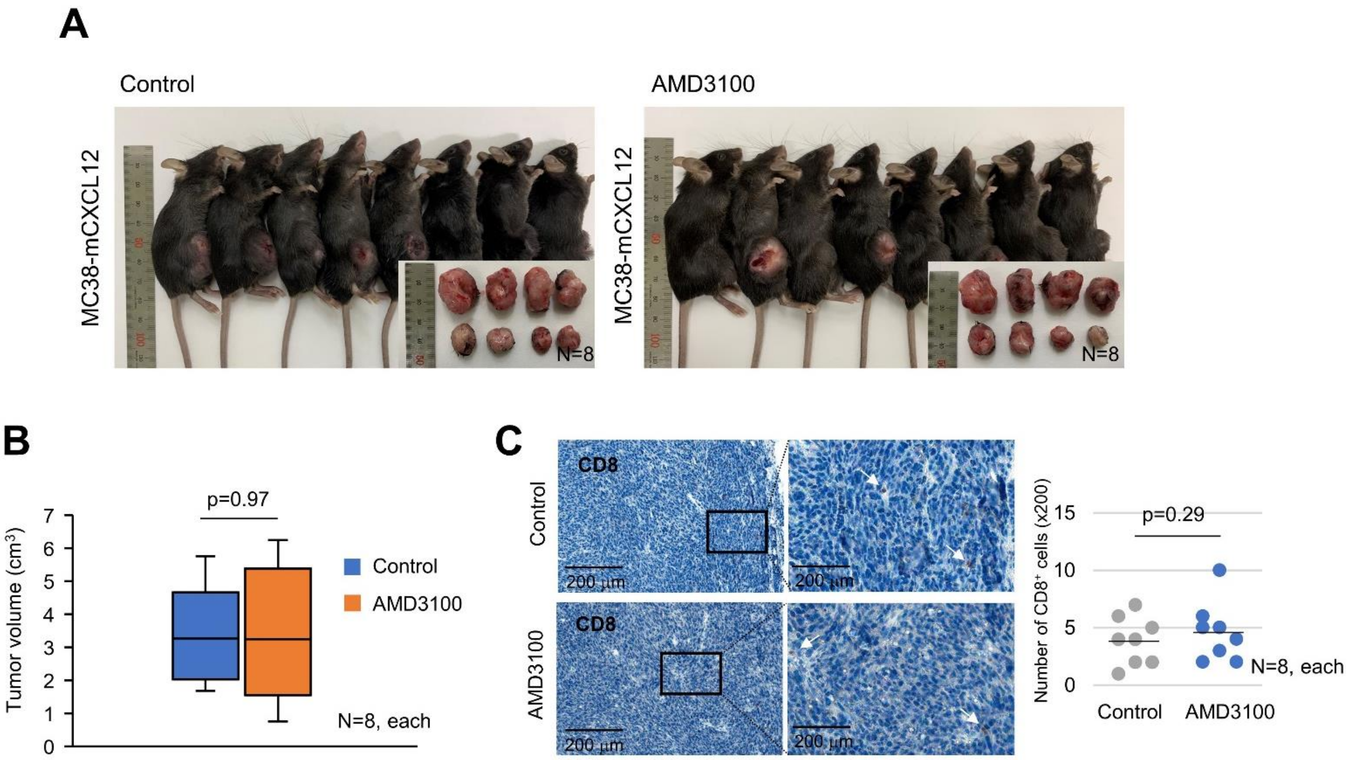

Figure S7. Tumor volume analysis in mouse colon cancer allograft model with mCXCL12 overexpressing MC38 cells after AMD3100 treatment. A) mCXCL12 overexpressing MC38 cells were transplanted into 7-week-old C57BL/6 mice. One week later, the mice were treated with AMD3100 (3 mg/kg) through intraperitoneal injection every day for two weeks. The mice were euthanized after three weeks, and the tumor size was analyzed. **B**) The final tumor volume was presented as a Box-and-Whisker diagram. The line inside the box is median. The top and the bottom of the box are the 75% and 25% percentile, respectively. Error bars on the whiskers represent minimum to maximum. **C**) To measure the number of tumor infiltrating T cells, three randomly selected areas of the tumor tissue per animal were photographed and then analyzed for $CD8^+$ cell infiltration; the results were averaged and presented as a dot graph. The *p* value was calculated by Mann–Whitney U test. N indicated the number of cases. The white arrow indicates the $CD8^+$ cells.

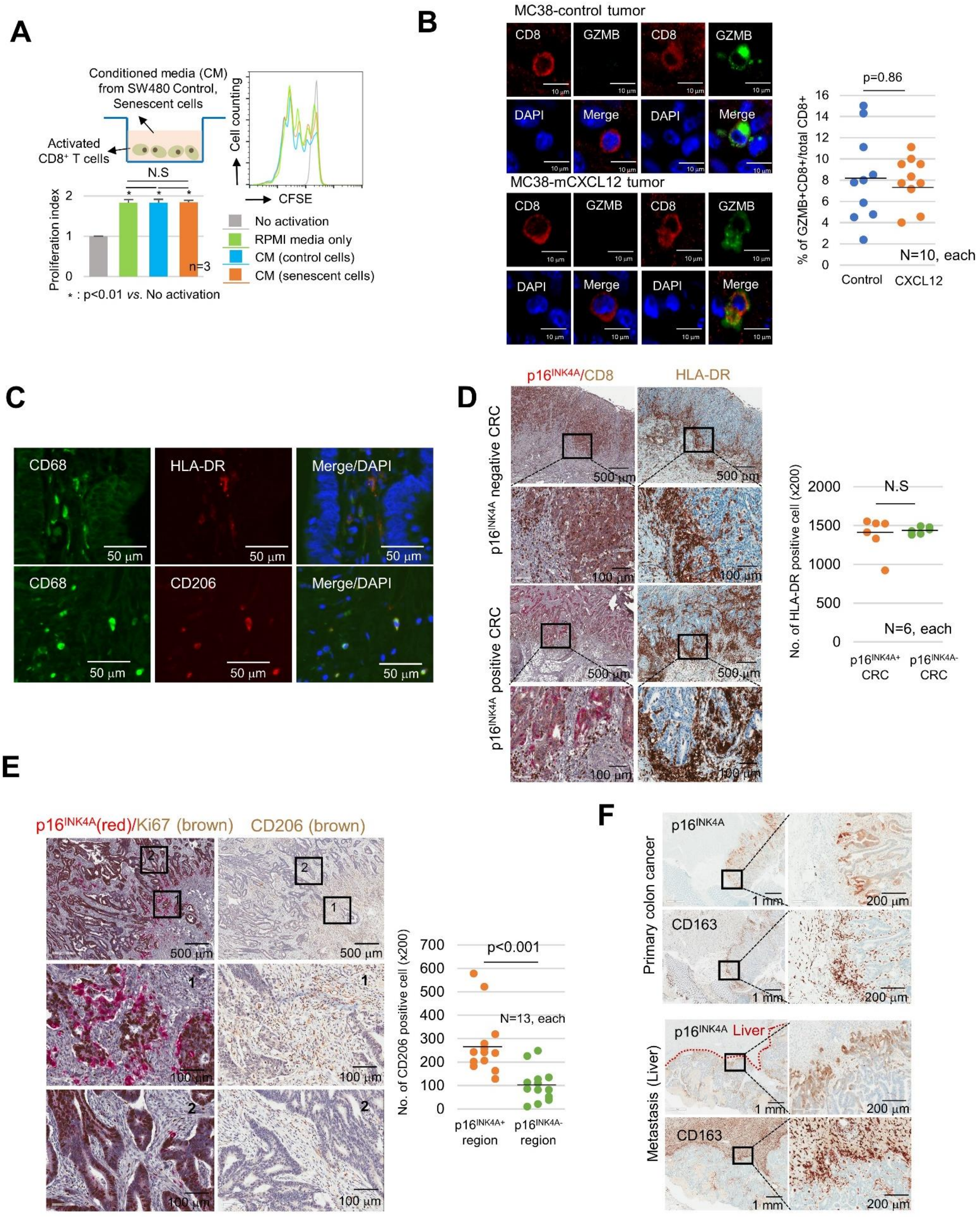
A
Conditioned media (CM) from SW480 Control, Senescent cells
Activated CD8+ T cells
Cell counting
CFSE
N.S
Proliferation index
n=3
No activation
RPMI media only
CM (control cells)
CM (senescent cells)
*: p<0.01 vs. No activation
B
MC38-control tumor
CD8
GZMB
DAPI
Merge
MC38-mCXCL12 tumor
10 μm
p=0.86
% of GZMB+CD8+/total CD8+
N=10, each
Control
CXCL12
C
CD68
HLA-DR
Merge/DAPI
CD206
50 μm
D
p16INK4A/CD8
HLA-DR
p16INK4A negative CRC
p16INK4A positive CRC
500 μm
100 μm
No. of HLA-DR positive cell (x200)
N.S
N=6, each
p16INK4A+ CRC
p16INK4A- CRC
E
p16INK4A(red)/Ki67 (brown)
CD206 (brown)
500 μm
100 μm
No. of CD206 positive cell (x200)
p<0.001
N=13, each
p16INK4A+ region
p16INK4A- region
F
Primary colon cancer
Metastasis (Liver)
p16INK4A
CD163
Liver
1 mm
200 μm

Figure S8. Characterization of macrophages associated with senescent tumor cells. A) T cell proliferation assay. CFSE-labelled $CD8^+$ T cell activation by anti-CD3/CD28 beads was performed using the CM from SW480 or SW480/ROS for 72 hours. Dilution of CFSE was measured by flow cytometry and proliferation index was subsequently calculated. The *p* value was calculated by one-way ANOVA and post hoc analysis. **B**) Granzyme B (GZMB)/CD8 double immunostaining was performed on the MC38-control and on the MC38-mCXCL12 overexpressing tumor (Figure 3F), and the percentage of double positive cells were counted and presented as a dot graph. **C**) CRC tissues were immunostained for CD206 or HLA-DR with CD68, respectively. **D**) Macrophage distribution analysis with HLA-DR in $p16^{INK4A}$ positive or negative CRC. Three randomly selected areas of the tumor tissue were photographed (×200) and then analyzed for HLA-DR positive cell. The results were averaged and then presented as a dot graph. **E**) CD206-positive cell distribution in $p16^{INK4A}$ positive CRC. Colon cancer tissues were serially dissected and stained with $p16^{INK4A}$, Ki67 and CD206. The '1' and '2' indicate the high magnification views of the original figure. Three randomly selected areas were photographed (×200) and then analyzed for CD206 positive cell. The results were averaged and then presented as a dot graph. **F**) Primary colon cancer and liver metastatic region were immunostained with $p16^{INK4A}$ and CD163. The dotted line indicates the cancer margin. The *p* value was calculated by Mann–Whitney U test (B, D, and, E). N indicated the number of cases.

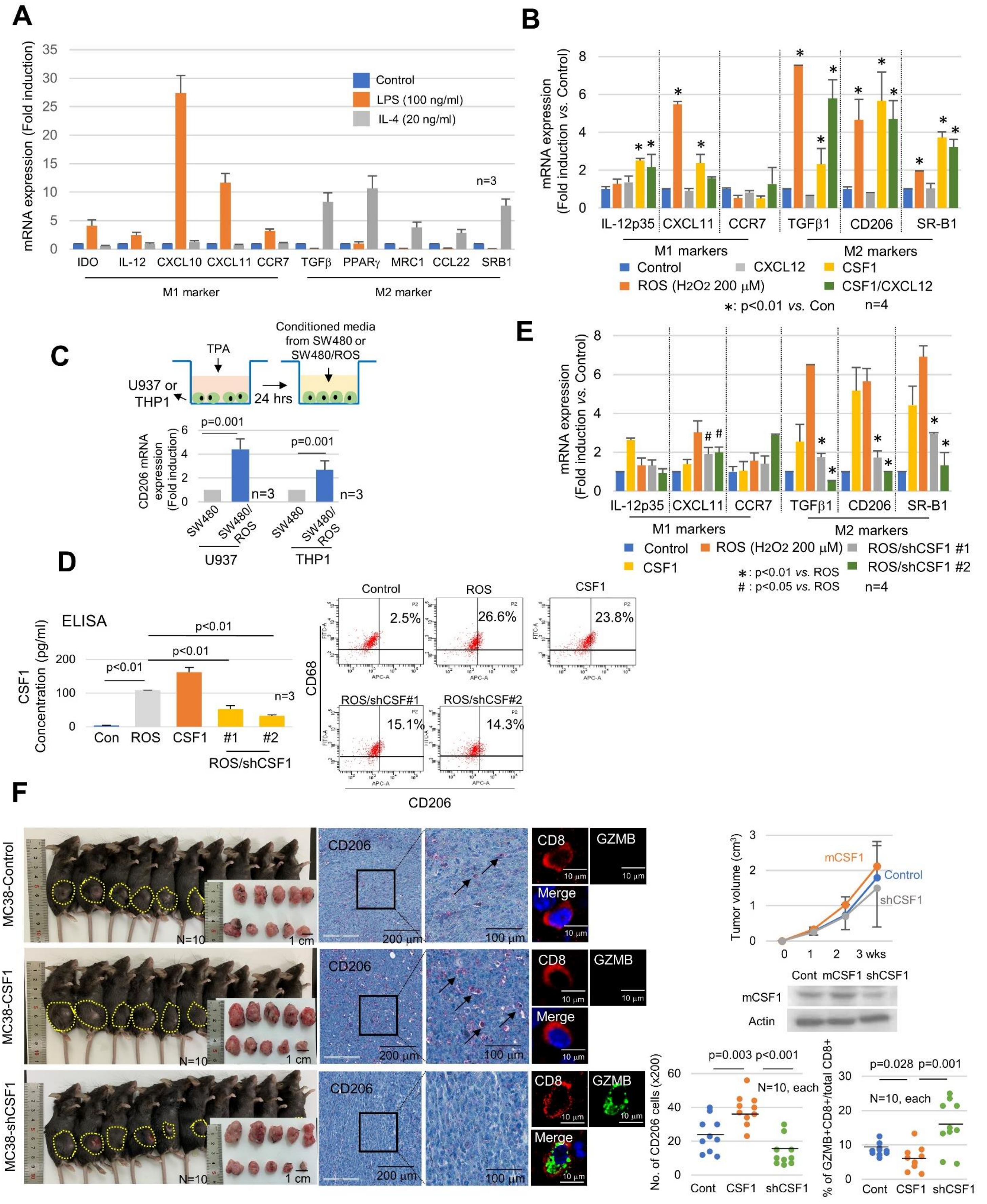

A
mRNA expression (Fold induction)
Control
LPS (100 ng/ml)
IL-4 (20 ng/ml)
n=3
IDO IL-12 CXCL10 CXCL11 CCR7 TGFβ PPARγ MRC1 CCL22 SRB1
M1 marker
M2 marker
B
mRNA expression (Fold induction vs. Control)
IL-12p35 CXCL11 CCR7 TGFβ1 CD206 SR-B1
M1 markers
M2 markers
Control
CXCL12
CSF1
ROS (H2O2 200 μM)
CSF1/CXCL12
*: p<0.01 vs. Con
n=4
C
TPA
U937 or THP1
24 hrs
Conditioned media from SW480 or SW480/ROS
CD206 mRNA expression (Fold induction)
p=0.001
p=0.001
n=3
n=3
SW480
SW480/ROS
SW480
SW480/ROS
U937
THP1
D
ELISA
CSF1 Concentration (pg/ml)
p<0.01
p<0.01
p<0.01
n=3
Con ROS CSF1 #1 #2
ROS/shCSF1
Control
ROS
CSF1
2.5%
26.6%
23.8%
ROS/shCSF#1
ROS/shCSF#2
15.1%
14.3%
CD68
CD206
E
mRNA expression (Fold induction vs. Control)
IL-12p35 CXCL11 CCR7 TGFβ1 CD206 SR-B1
M1 markers
M2 markers
Control
ROS (H2O2 200 μM)
ROS/shCSF1 #1
CSF1
ROS/shCSF1 #2
*: p<0.01 vs. ROS
#: p<0.05 vs. ROS
n=4
F
MC38-Control
MC38-CSF1
MC38-shCSF1
N=10
1 cm
CD206
200 μm
100 μm
CD8
GZMB
Merge
10 μm
Tumor volume (cm3)
mCSF1
Control
shCSF1
0 1 2 3 wks
Cont mCSF1 shCSF1
mCSF1
Actin
No. of CD206 cells (x200)
p=0.003 p<0.001
N=10, each
Cont CSF1 shCSF1
% of GZMB+CD8+/total CD8+
p=0.028 p=0.001
N=10, each
Cont CSF1 shCSF1

Figure S9. Senescent tumor cells polarize monocytes to M2 macrophages. A) LPS and IL4 polarized monocytes to M1 and M2 macrophages, respectively. Isolated primary monocytes were treated with LPS (100 ng/ml) or IL4 (20 ng/ml) for three days and M1 and M2 macrophage markers were analyzed by real time PCR. **B**) Isolated primary monocytes were cocultured with SW480 (control, H_2O_2 treated, CSF1 overexpressing, and CXCL12 overexpressing) for six days and then analyzed for macrophage markers by real time PCR. **C**) Senescent tumor cells induced M2 macrophage differentiation in U937 and THP1 cells. U937 or THP1 cells were treated with TPA for 24 hours, and then the media were replaced with CM from SW480 or H_2O_2 treated SW480 cells. U937 and THP1 cells were maintained for 3 days and then analyzed for CD206 expression by real time PCR. The *p* value was calculated using Mann–Whitney U test. **D, E**) Two kinds of shCSF1 lentivirus infected ROS treated SW480 cells were cocultured with isolated primary monocytes for six days. The cells were analyzed for CD206 expression by FACS (D) and the macrophage markers by real time PCR (E). **F**) CSF1 regulate $CD8^+$ T cell activation in the animal model. Control, mouse CSF1 overexpressing, or mouse CSF1 knockdown MC38 cells ($1x10^6$) were transplanted into female C57BL/6 mice and maintained for three weeks with the tumor growth analysis (upper right panel). Tumor volume was presented as mean ± SD with a line graph. The mice were euthanized, and macrophage differentiation was analyzed with CD206 immunohistochemistry. $CD8^+$ T cell activation was analyzed with GZMB and presented as a percentage of $GZMB^+/CD8^+$ per total $CD8^+$. CSF1 protein expression was analyzed by western blot. The *p* value was calculated using Kruskall Wallis test (B, D, E, and F). N and n indicated the number of cases and independent experiments, respectively.

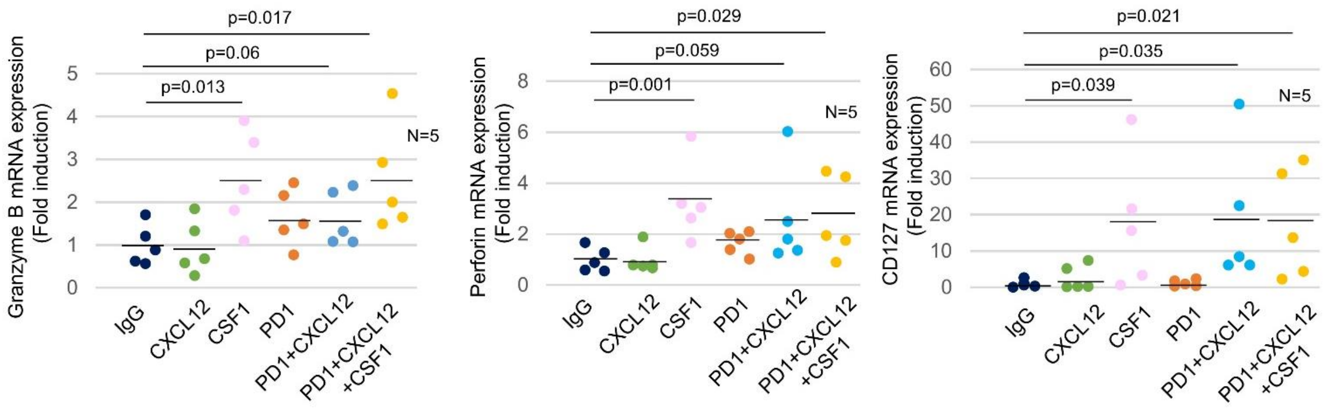

Figure S10. Analysis of gene expression associated activated T cell. Granzyme B, perforin, and CD127 mRNA expression was analyzed in tumors from mCXCL12 overexpressing MC38 cells bearing mice (Figure 6C) by real time PCR and presented as fold induction compared with those of IgG injected mice.

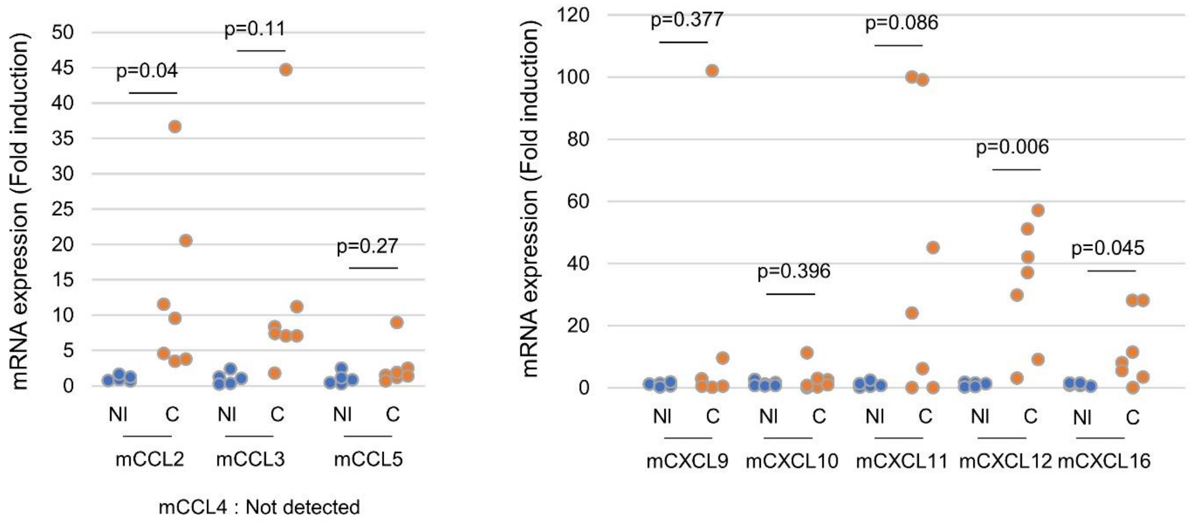

Figure S11. T cell Chemokine expression in AOM/DSS-induced mouse CRC. mRNA expression of T cell chemokines was analyzed in normal colon (Nl, N=5) and AOM/DSS induced mouse CRC (C, N=7) by real time PCR and presented as fold induction compared with those of normal colon.

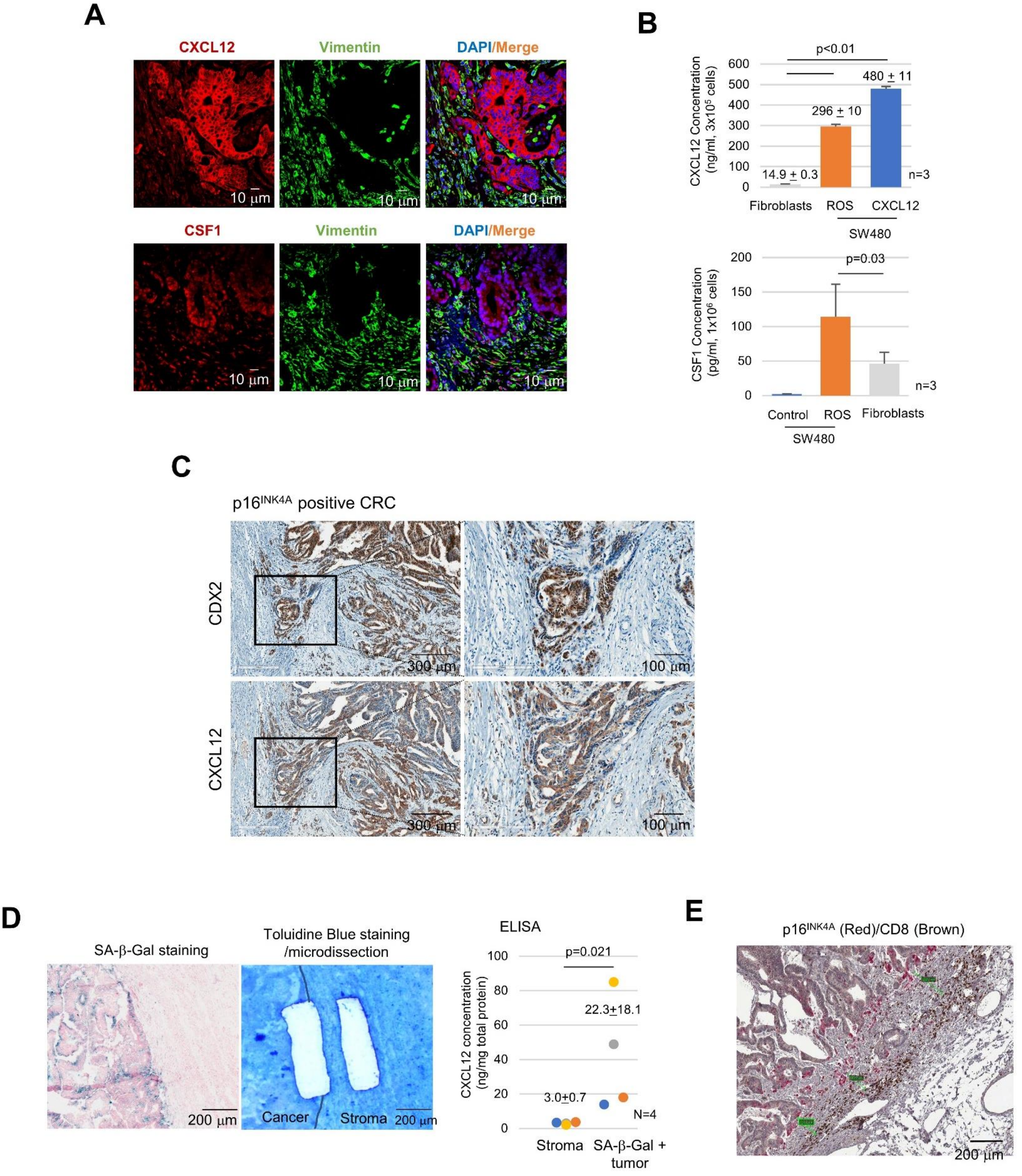
A
CXCL12
Vimentin
DAPI/Merge
CSF1
Vimentin
DAPI/Merge
10 µm
B
p<0.01
CXCL12 Concentration (ng/ml, 3x10^5 cells)
14.9 ± 0.3
296 ± 10
480 ± 11
n=3
Fibroblasts
ROS
CXCL12
SW480
p=0.03
CSF1 Concentration (pg/ml, 1x10^6 cells)
n=3
Control
ROS
Fibroblasts
SW480
C
p16INK4A positive CRC
CDX2
CXCL12
300 µm
100 µm
D
SA-β-Gal staining
Toluidine Blue staining /microdissection
200 µm
Cancer
Stroma
ELISA
p=0.021
CXCL12 concentration (ng/mg total protein)
22.3±18.1
3.0±0.7
N=4
Stroma
SA-β-Gal + tumor
E
p16INK4A (Red)/CD8 (Brown)
200 µm

Figure S12. Expression of CXCL12 and the distance between senescent tumor cells and $CD8^+$ T cells in $p16^{INK4A}$ positive CRC. A) Analysis of CXCL12 and CSF1 expression in the cancer epithelial cells and stromal cells (fibroblasts) of $p16^{INK4A}$ positive CRC. Double immunostaining highlighted CXCL12 or CSF1 expression in cancer (vimentin negative) and stromal (vimentin positive) cells (left panel). **B**) ELISA analysis of CXCL12 or CSF1 in isolated fibroblast and ROS induced SW480 senescent cells. C) $p16^{INK4A}$ positive CRC was dissected serially and stained with CDX2 and CXCL12, respectively. D) ELISA analysis of CXCL12 in the $p16^{INK4A}$ positive tumor epithelial region and adjacent stromal region individually isolated by microdissection (N=4). E) The distance between senescent tumor cells (red) and $CD8^+$ T cells (brown). Three points were measured using Aperio ImageScope viewing software (Leica Biosystems). The *p* value was calculated using Kruskall Wallis test (B) or Mann–Whitney U test (D). N and n indicated the number of cases and independent experiments, respectively.

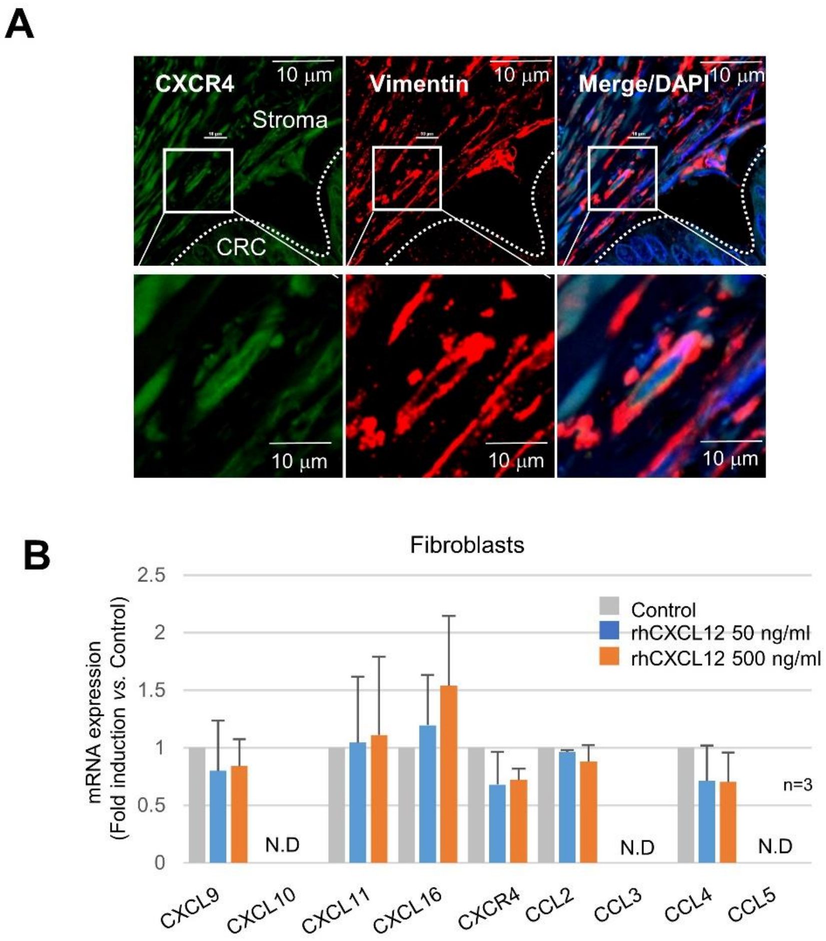

Figure S13. CXCR4 expression in stromal fibroblasts. A) CRC was double stained with CXCR4 (green) and vimentin (red). **B**) Fibroblasts were treated with a low concentration (50 ng/ml) or high concentration (500 ng/ml) of rhCXCL12 for 24 hours and then analyzed CXCL9, CXCL10, CXCL11, CXCL16, CCL2, CCL4, and CXCR4 mRNA expression by real time PCR.

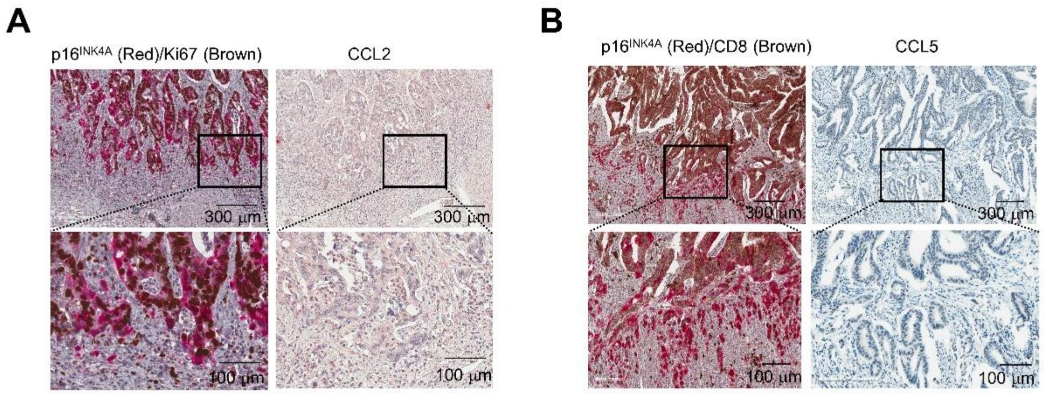

Figure S14. CCL2 and CCL5 expression in CRC. A, B) p16^{INK4A} positive CRC was dissected serially and stained with p16^{INK4A}/CCL2 (A) and p16^{INK4A}/CCL5 (B), respectively.

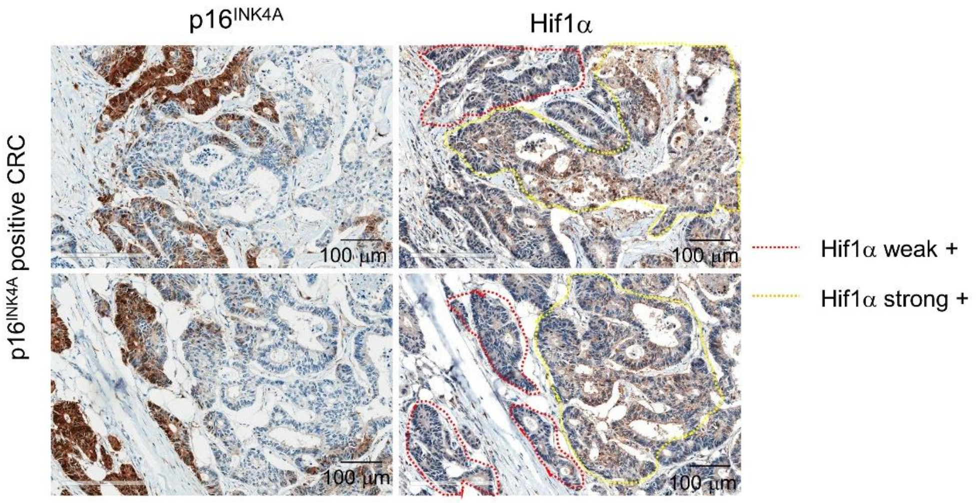

Figure S15. Hif1α expression in CRC. p16^{INK4A} positive CRC was dissected serially and stained with p16^{INK4A} and Hif1α, respectively. Red and yellow dot line indicate Hif1α weak and strong stained area, respectively.

Table S1. Clinicopathological characteristics of patients according to p16 INK4A expression

Characteristics	All cases (n=130)					MSS cases (n=120)				
	p16 INK4A expression (grade), *N* (%)				*p*	p16 INK4A expression (grade), *N* (%)				*p*
	**0**	**1**	**2**	**3**		**0**	**1**	**2**	**3**	
Age (years)										
< 60	4 (22.2)	12 (35.3)	18 (46.2)	14 (35.9)	0.369	4 (22.2)	10 (34.5)	18 (47.4)	13 (37.1)	0.257
≥ 60	14 (77.8)	22 (64.7)	21 (53.8)	25 (64.1)		14 (77.8)	19 (65.5)	20 (52.6)	22 (62.9)	
Gender										
Female	7 (38.9)	10 (29.4)	22 (56.4)	18 (46.2)	0.13	7 (38.9)	8 (27.6)	21 (55.3)	17 (48.6)	0.144
Male	11 (61.1)	24 (70.6)	17 (43.6)	21 (53.8)		11 (61.1)	21 (72.4)	17 (44.7)	18 (51.4)	
Tumor location										
Right	2 (11.1)	5 (14.7)	8 (20.5)	6 (15.4)	0.818	2 (11.1)	4 (13.8)	7 (18.4)	6 (17.1)	0.508
Left	16 (88.9)	29 (85.3)	31 (79.5)	33 (84.6)		16 (88.9)	25 (86.2)	31 (81.6)	29 (82.9)	
Tumor size										
< 4 cm	5 (27.8)	9 (26.5)	11 (28.2)	10 (25.6)	1.000	5 (27.8)	8 (27.6)	11 (28.9)	9 (25.7)	0.883
≥ 4 cm	13 (72.2)	25 (73.5)	28 (71.8)	29 (74.4)		13 (72.2)	21 (72.4)	27 (71.1)	26 (74.3)	
T stage										
pT1, 2	3 (16.7)	7 (20.6)	2 (5.1)	3 (7.7)	0.088	3 (16.7)	6 (20.7)	2 (5.3)	3 (8.6)	0.132
pT3, 4	15 (83.3)	27 (79.4)	37 (94.9)	36 (92.3)		15 (83.3)	23 (79.3)	36 (94.7)	32 (91.4)	
N stage										
pN0, N1	14 (77.8)	27 (79.4)	27 (69.2)	20 (51.3)	0.011	14 (77.8)	22 (75.9)	26 (68.4)	18 (51.4)	0.025
pN2	4 (22.2)	7 (20.6)	12 (30.8)	19 (48.7)		4 (22.2)	7 (24.1)	12 (31.6)	17 (48.6)	
M stage										
M0	16 (88.9)	29 (85.3)	31 (79.5)	30 (76.9)	0.210	16 (88.9)	24 (82.8)	30 (78.9)	27 (77.1)	0.289
M1	2 (11.1)	5 (14.7)	8 (20.5)	9 (23.1)		2 (11.1)	5 (17.2)	8 (21.1)	8 (22.9)	
TNM stage										
I, II	12 (66.7)	18 (52.9)	15 (38.5)	14 (35.9)	0.017	12 (66.7)	14 (48.3)	14 (36.8)	14 (40.0)	0.064
III, IV	6 (33.3)	16 (47.1)	24 (61.5)	25 (64.1)		6 (33.3)	15 (51.7)	24 (63.2)	21 (60.0)	
Tumoral immune infiltration [a]										
Weak	1 (5.6)	3 (8.8)	11 (28.2)	21 (53.8)	<0.001	1 (5.6)	3 (10.3)	11 (28.9)	17 (48.6)	<0.001
Moderate, strong	17 (94.4)	31 (91.2)	28 (71.8)	18 (46.2)		17 (94.4)	26 (89.7)	27 (71.1)	18 (51.4)	
Lymphatic invasion										
No	12 (66.7)	18 (52.9)	22 (56.4)	17 (43.6)	0.399	12 (66.7)	13 (44.8)	21 (55.3)	14 (40.0)	0.243
Yes	6 (33.3)	16 (47.1)	17 (43.6)	22 (56.4)		6 (33.3)	16 (55.2)	17 (44.7)	21 (60.0)	
Venous invasion										
No	16 (88.9)	33 (97.1)	34 (87.2)	36 (92.3)	0.429	16 (88.9)	28 (96.6)	33 (86.8)	32 (91.4)	0.531
Yes	2 (11.1)	1 (2.9)	5 (12.8)	3 (7.7)		2 (11.1)	1 (3.4)	5 (13.2)	3 (8.6)	
Perineural invasion										
No	15 (83.3)	30 (88.2)	26 (66.7)	24 (61.5)	0.030	15 (83.3)	25 (86.2)	25 (65.8)	21 (60.0)	0.054
Yes	3 (16.7)	4 (11.8)	13 (33.3)	15 (38.5)		3 (16.7)	4 (13.8)	13 (34.2)	14 (40.0)	

N: number, MSS: microsatellite stable

P values were calculated using the $\chi 2$ test

[a]defined as follows: weak = presence of immune cells occupying < 10 % of the cancer tissue, moderate = presence of immune cells occupying 10 to 30 % of the cancer tissue, strong = immune cells occupying > 30% of the cancer tissue.

Table S2. Distribution of RAS/BRAF, PIK3CA, TP53, and APC mutations according to p16 ^{INK4A} expression

Mutated genes	p16 ^{INK4A} expression (grade), *N* (%)				*p*
	**0**	**1**	**2**	**3**	
RAS/BRAF[a]	2/6 (33.3)	4/13 (30.8)	13/24 (54.2)	11/21 (52.4)	0.454
PIK3CA	1/4 (25.0)	1/6 (16.7)	1/13 (7.7)	4/14 (28.6)	0.535
TP53	3/4 (75.0)	2/6 (33.3)	10/13 (76.9)	9/14 (64.3)	0.315
APC	2/4 (50.0)	5/6 (83.3)	6/13 (46.2)	8/14 (57.1)	0.458

N: number
P values were calculated using the $\chi 2$ test
[a]Including *KRAS* (62 cases), *NRAS* (1 case), and *BRAF* (1 case) mutations.

Supplementary materials

Movie S1. Jurkat T cell migration in the presence of rhCXCL12. CXCL12 chemo-gradient was developed using μ-Slide and analyzed Jurkat T cell migration. The live cell imaging was recorded for 24 hrs on a JuLi stage system operating on a CO_2 incubator.

Movie S2. Jurkat T cell migration in the presence of CXCL12 expressing cells. CXCL12 chemogradient was developed by CXCL12 overexpressing SW480 cells in μ-Slide and then analyzed T cell migration. The live cell imaging was recorded for 8 hrs on a JuLi stage system operating on a CO_2 incubator.

Movie S3. Lamellipodia formation in low concentration of rhCXCL12. Jurkat T cells were treated with 0, 50 or 1,000 ng/ml rhCXCL12 in μ-Slide and analyzed lamellipodia formation with SiR-actin staining. The live cell imaging was recorded for 10 min on confocal microscope (Nikon) with a x60 oil objective and a temperature-controlled chamber (37°C, 5% CO_2).
